# Data gaps and outliers distort critical-slowing-down-based resilience indicators

**DOI:** 10.1126/sciadv.aee1916

**Published:** 2026-03-13

**Authors:** Teng Liu, Andreas Morr, Sebastian Bathiany, Lana L. Blaschke, Zhen Qian, Chan Diao, Taylor Smith, Niklas Boers

**Affiliations:** ^1^ Munich Climate Center and Earth System Modelling Group, Department of Aerospace and Geodesy, TUM School of Engineering and Design, Technical University of Munich, Munich 80333, Germany.; ^2^ Potsdam Institute for Climate Impact Research, Potsdam 14473, Germany.; ^3^ School of Systems Science and Institute of Nonequilibrium Systems, Beijing Normal University, Beijing 100875, China.; ^4^ Department of Mathematics, School of Computation, Information and Technology, Technical University of Munich, Garching 85748, Germany.; ^5^ Faculty of Geographical Science, Beijing Normal University, Beijing 100875, China.; ^6^ Institute of Geosciences, Universität Potsdam, Potsdam 14476, Germany.

## Abstract

The resilience of natural systems, such as climate or ecosystems, is increasingly threatened by anthropogenic pressures, making it essential to quantify resilience changes before abrupt and irreversible regime shifts occur. Widely used data-driven resilience indicators based on variance and autocorrelation detect “critical slowing down,” a signature of decreasing stability and possible impending critical transitions in dynamical systems with alternative equilibria. However, the interpretation of these indicators is complicated by common data issues such as missing values and outliers, whose effects remain poorly understood. Here, we develop a general mathematical framework that rigorously characterizes the statistical dependency between variance- and autocorrelation-based resilience indicators, revealing that their agreement is fundamentally driven by the time series’ initial data point. Using synthetic and empirical data, we demonstrate that missing values substantially weaken the agreement of resilience indicators, while outliers introduce systematic biases that lead to overestimation of resilience based on temporal autocorrelation. Our results provide a necessary and rigorous foundation for preprocessing strategies and accuracy assessments across the growing number of disciplines that use empirical data to infer changes in system resilience.

## Teaser

Data gaps and outliers distort the statistical indicators used to detect loss of resilience in natural systems.

## INTRODUCTION

Resilience is a fundamental property of dynamical systems with stable equilibrium states, describing their ability to absorb disturbances and recover from perturbations without undergoing fundamental shifts in structure or function (*1*, *2*). In systems with strong nonlinear interactions and resulting positive feedbacks, small perturbations may trigger irreversible transitions if resilience is low (*3*). This behavior is observed across diverse domains, from climate systems and financial markets to biological networks and ecosystems (*4*–*7*).

Monitoring the resilience of nonlinear natural systems, and in particular Earth system components and ecosystems, is crucial, particularly in the face of intensifying anthropogenic pressures. Several major climate subsystems have been suggested to be at risk of critical transitions in response to anthropogenic forcing (*4*, *8*, *9*), which can also trigger abrupt responses in other parts of the Earth system, such as ecosystems. More broadly, changes in resilience can cascade across coupled natural and human systems, with consequences for biodiversity, food and water security, financial stability, and human well-being (*10*–*12*). Ecosystem resilience, in particular, plays a fundamental role in maintaining biodiversity, natural carbon sinks, and other essential ecosystem services (*13*). However, anthropogenic climate change and human activities are increasingly eroding this resilience, potentially pushing many ecosystems toward critical thresholds (*14*, *15*). A prominent example is the Amazon rainforest, where positive atmosphere-vegetation feedbacks and repeated disturbances such as droughts, wildfires, and deforestation are weakening the ability of forests to maintain their current state, raising concerns about an abrupt shift to a drier, less biodiverse, and low-tree-cover Amazon (*16*–*22*). Similar decreases in resilience also threaten coral reefs, boreal forests, and tundra systems, where external shocks can lead to long-term transformation in ecosystems (*8*).

Understanding and quantifying resilience is essential for predicting and mitigating abrupt regional shifts. Empirical approaches typically define resilience as the rate of recovery from external disturbances (*23*, *24*). Consequently, measuring resilience directly requires either controlled experiments involving artificial perturbations or natural observations of strong external disturbances (*25*). An alternative approach is based on the fluctuation-dissipation theorem, which states that the rate at which a system returns to equilibrium after a disturbance can be inferred from its internal variability (*26*). This allows one to leverage statistical indicators to infer resilience from small, natural fluctuations within a system (*27*). Key statistical measures derived from this principle include variance (representing the magnitude of fluctuations) and lag-one autocorrelation (AC1, a measure of the system’s memory) (*28*, *29*). In systems that are known a priori to exhibit bistability, with another stable fixed point beyond the current basin of attraction, the recovery rate decreases as the system approaches a critical bifurcation threshold, a phenomenon referred to as critical slowing down (CSD). Consequently, both variance and AC1 tend to increase (*30*, *31*). These CSD indicators have been widely used as proxies for resilience changes and as early warning signals of abrupt change in a wide range of systems, including ecosystems (*15*, *27*, *32*, *33*), climate systems (*34*–*36*), paleoclimate records (*37*–*39*), and other complex systems such as biological systems (*40*, *41*) and psychological dynamics (*42*).

Despite broad applicability, CSD-based resilience indicators face two fundamental challenges when applied to real-world systems. First, their theoretical validity depends on the underlying dynamical framework, specifically, on whether the system can be locally approximated by a stable fixed point under noise forcing, where recovery from perturbations can be meaningfully described as CSD (*43*–*45*). Deviations from these assumptions, such as unfavorable chaotic dynamics (*44*) or a benign widening of potential wells (*43*, *45*), can lead to misinterpretation of rising variance or AC1 as signs of instability. Second, even when the theoretical framework is appropriate, practical data limitations can strongly affect CSD indicator behavior (*36*, *46*–*48*). For instance, multisensor data with varying signal-to-noise ratios may result in nonstationary higher-order statistical properties that distort variance and AC1 and can lead to erroneous resilience estimates (*46*). In addition, time-correlated noise can introduce spurious changes in both variance and AC1 that are unrelated to actual changes in resilience (*34*, *49*, *50*). To gauge the influence of such issues, Smith and Boers (*51*) proposed jointly analyzing $\lambda_{\text{Var}}$ (a variance-based indicator) and $\lambda_{\text{AC}1}$ (an AC1-based indicator), arguing that their agreement provides a justification for their use. Deviations between these indicators were suggested as an uncertainty metric on the underlying modelling assumptions (*51*–*53*). Applying this framework to global vegetation dynamics, they found that the consistency between $\lambda_{\text{Var}}$ and $\lambda_{\text{AC}1}$ varies with biomass levels, with lower agreement observed in high-biomass regions such as tropical forests. However, a formal mathematical explanation for the relationship between $\lambda_{\text{Var}}$ and $\lambda_{\text{AC}1}$ remains absent, and the mechanism underlying the link between $\lambda_{\text{Var}}$-$\lambda_{\text{AC}1}$ agreement and biomass levels is unclear.

In this study, we present a general analytical framework for understanding the relationship between the two most widely used resilience indicators $\lambda_{\text{Var}}$ and $\lambda_{\text{AC}1}$. We derive an analytical expression that characterizes their statistical dependence and reveals a fundamental sensitivity to the initial conditions of the time series that $\lambda_{\text{Var}}$ and $\lambda_{\text{AC}1}$ are computed on. To investigate the impact of realistic data imperfections, we generate synthetic time series with controlled missing values and outliers and assess their impact on the agreement between the resilience indicators. We then use global vegetation as an illustrative example, supported by satellite-derived vegetation indices across different land-cover types. Our results show that missing values, a particular problem in high-biomass regions such as rainforests, substantially weaken indicator agreement, while outliers can systematically bias $\lambda_{\text{AC}1}$ downward, leading to potential overestimation of resilience.

## RESULTS

### Statistical dependence of resilience indicators

We begin by reexamining the definition of the two CSD-based resilience indicators, $\lambda_{\text{AC}1}$ and $\lambda_{\text{Var}}$. Both are derived from an observed time series $X=\left\{X_{1},\ldots ,X_{N}\right\}$, which represents the deviations of a system variable from its equilibrium state. The autocorrelation-based indicator quantifies the restoring rate inferred from the system’s short-term memory, as

$$\lambda_{\text{AC}1}=\text{log}\left(\hat{\text{AC}1}\right)$$ (1)

where the empirical estimator of AC1 is: $\hat{\text{AC}1}=\left(\sum_{i=1}^{N-1}X_{i}X_{i+1}\right)/\left(\sum_{i=1}^{N}X_{i}^{2}\right)$. This quantity measures the linear dependence between successive observations. Larger (less negative) values of $\lambda_{\text{AC}1}$ indicate stronger short-term memory and slower recovery from perturbations, corresponding to lower resilience. The variance-based indicator captures the same underlying restoring rate inferred instead from the size of the system’s fluctuations, as

$$\lambda_{\text{Var}}=\frac{1}{2}\text{log}\left(1-\frac{{\hat{\sigma }}_{\varepsilon }^{2}}{\text{Var}\left[X\right]}\right)$$ (2)

where $\text{Var}\left[X\right]=\left(\sum_{i=1}^{N}X_{i}^{2}\right)/N$ is the empirical variance of the time series, and ${\hat{\sigma }}_{\varepsilon }^{2}$ is the variance of the residual noise term obtained from fitting an order-one autoregressive [AR(1)] model, i.e., $X_{i+1}=\hat{\text{AC}1}X_{i}+{\hat{\sigma }}_{\varepsilon }\varepsilon_{i}$. The residual variance is computed in an ordinary linear regression framework as

$${\hat{\sigma }}_{\varepsilon }^{2}=\frac{1}{N-1}\sum_{i=1}^{N-1}{\left(X_{i+1}-\hat{\text{AC}1}\cdot X_{i}\right)}^{2}$$ (3)

Therefore, ${\hat{\sigma }}_{\varepsilon }$ quantifies the magnitude of random fluctuations not explained by the linear dependence between successive data points. Because this quantity explicitly depends on $\hat{\text{AC}1}$, the two indicators are inherently linked. Substituting Eq. 3 into Eq. 2 yields a direct functional relationship between $\lambda_{\text{AC}1}$ and $\lambda_{\text{Var}}$ (see Methods for derivation)

$$\begin{array}{c} \lambda_{\text{Var}}=\\ \frac{1}{2}\text{log}\left\{\underset{\text{first term}}{\underbrace{1-\frac{N}{N-1}\left[1-\text{exp}\left(2\lambda_{\text{AC}1}\right)\right]}}+\underset{\text{second term}}{\underbrace{\frac{1}{N-1}\frac{X_{1}^{2}+\text{exp}\left(2\lambda_{\text{AC}1}\right)X_{N}^{2}}{\text{Var}\left[X\right]}}}\right\} \end{array}$$ (4)

We note that, in the absence of the second term inside the logarithm, Eq. 4 simplifies to a universal relationship that is independent of any specific realization of the time series

$$\lambda_{\text{Var}}=\frac{1}{2}\text{log}\left\{1-\frac{N}{N-1}\left[1-\text{exp}\left(2\lambda_{\text{AC}1}\right)\right]\right\}$$ (5)

This expression serves as a lower bound for the relationship between $\lambda_{\text{Var}}$ and $\lambda_{\text{AC}1}$, as the second term in Eq. 4 is nonnegative. While the second term captures the influence of specific realizations of the time series, this influence depends only on the relative amplitude of the first and last data points (normalized by the average amplitude). Since $\lambda_{\text{AC}1}$ is negative, $\text{exp}\left(2\lambda_{\text{AC}1}\right)X_{N}^{2}$ is typically negligible compared to $X_{1}^{2}$, rendering the second term primarily determined by $X_{1}^{2}/\text{Var}\left[X\right]$ (see the Supplementary Materials). Therefore, Eq. 4 confirms not only that the two resilience indicators, $\lambda_{\text{Var}}$ and $\lambda_{\text{AC}1}$, are not statistically independent but also that their relationship is largely determined by the relative amplitude of the first data point rather than by the underlying system dynamics.

**Fig. 1.**
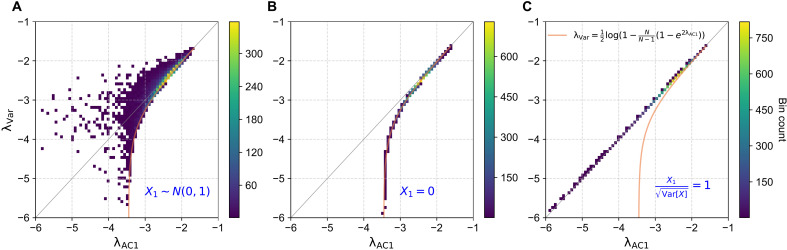
Relationship between resilience indicators $\lambda_{\text{Var}}$ and $\lambda_{\text{AC}1}$ under different initial conditions. For each condition we generate n=10,000 time series from an AR(1) process, each of length 1000. Three scenarios are considered: (**A**) The first data point is unchanged, i.e., $X_{1}∼N\left(0,1\right)$; (**B**) the first data point is artificially set as $X_{1}=0$; and (**C**) the first data point is set to satisfy $X_{1}/\sqrt{\text{Var}\left[X\right]}=1$. The orange curve represents the lower bound of the distribution, as characterized by the universal function given in Eq. 5.

To numerically confirm the relationship established in Eq. 4, we generate a total of 10,000 time series from an AR(1) process with parameters $\alpha =e^{-2.5}$ and $\sigma_{\varepsilon }=1$ (see Methods). For all synthetic experiments, we generated time series of length *N* = 1000, consistent with the typical record length of widely used satellite and observational datasets [e.g., Moderate Resolution Imaging Spectroradiometer (MODIS) or Global Inventory Modeling and Mapping Studies normalized difference vegetation index (NDVI)]. We then modify the first data point of each series to investigate its influence on the relationship between the resilience indicators $\lambda_{\text{Var}}$ and $\lambda_{\text{AC}1}$. Specifically, we consider three scenarios: (i) The baseline scenario did not involve modifications, with the amplitude of the first data point following a normal distribution, i.e., $X_{1}∼N\left(0,1\right)$, representing expectations for a random initial measurement chosen from a stationary dynamic real-world system. As shown in Fig. 1A, the relationship between $\lambda_{\text{Var}}$ and $\lambda_{\text{AC}1}$ lies above the universal lower bound (the orange curve). This theoretical lower bound, described by Eq. 5, provides a quantitative constraint on the relationship between $\lambda_{\text{Var}}$ and $\lambda_{\text{AC}1}$.

(ii) The first data point is set to zero, i.e., $X_{1}=0$. Here, the second term on the right-hand side of Eq. 4 almost vanishes, causing the relationship between $\lambda_{\text{Var}}$ and $\lambda_{\text{AC}1}$ to follow the universal lower bound (Eq. 5). Consequently, as shown in Fig. 1B, data points from different time series collapse onto the orange curve.

(iii) The relative amplitude of the first data point is set to unity, i.e., $X_{1}/\sqrt{\text{Var}\left[X\right]}=\pm 1$, representing the statistically expected situation in a stationary process. Under this constraint, Eq. 4 predicts an approximate equality between $\lambda_{\text{Var}}$ and $\lambda_{\text{AC}1}$ ($\lambda_{\text{Var}}\approx \lambda_{\text{AC}1}$), resulting in a clustering of data points along the identity line in the $\lambda_{\text{Var}}-\lambda_{\text{AC}1}$ scatter plot (Fig. 1C).

The dependence on $X_{1}$ arises from the different sampling schemes of the two estimators. The AC1-based estimator uses N−1 pairs ${\left\{\left(X_{i},X_{i+1}\right)\right\}}_{i=1}^{N-1}$, while the variance-based estimator uses *N* single observations ${\left\{X_{i}\right\}}_{i=1}^{N}$. Because the AC1 calculation lacks pairs at the beginning and end of the record, its residual sum of squares includes endpoint corrections, which algebraically lead to the $X_{1}$ and $X_{N}$ terms in Eq. 4, reflecting the unequal treatment of boundary values by the two estimators. Although these corrections are multiplied by 1/(N−1), their contribution remains nonnegligible for realistic record lengths ($N\leq 10^{3}$), especially under high-resilience conditions. The three scenarios above demonstrate that, in principle, a 1:1 relationship of $\lambda_{\text{Var}}$ and $\lambda_{\text{AC}1}$ can be expected, such as in the limit of an infinite number of data points *N*, or when taking the mean value across a huge number of realizations, since Eq. 4 reduces to $\lambda_{\text{Var}}=\lambda_{\text{AC}1}$ when N→∞. In finite samples, however, individual realizations inevitably exhibit scatter along the $\lambda_{\text{Var}}$ dimension, as illustrated in Fig. 1A. This scatter becomes more pronounced the shorter the record, typical of observational datasets and time-window-based analyses, because the boundary effect of the first data point strengthens as *N* decreases. As shown in fig. S1 (N=200, 500, 2000, and 4000), the effect intensifies markedly for shorter time series, in line with the finite-sample scaling predicted by Eq. 4.

While Fig. 1A is based on time series generated by an AR(1) process, it is important to note that the underlying relationship is broadly applicable: Eq. 4 holds for any gapless time series, irrespective of the specific data-generating mechanism. In other words, the same statistical dependence between variance- and AC1-based indicators emerges even when the time series is not linked to any underlying resilience or stabilization dynamics (fig. S2 for time series of random values). In summary, although $\lambda_{\text{Var}}$ and $\lambda_{\text{AC}1}$ are computed using the entire time series, their agreement is predominantly influenced by the first data point. Modifying only this initial value can substantially alter the relationship between the two indicators, even if all other properties of the time series remain unchanged. This sensitivity highlights the need for caution when interpreting strong correlations between these resilience indicators as sufficient evidence supporting the applicability of CSD analyses.

### Missing values undermine the agreement of resilience indicators

The statistical dependence between $\lambda_{\text{Var}}$ and $\lambda_{\text{AC}1}$, as expressed in Eq. 4, relies on the assumption that the time series contains no missing values (or data gaps). However, this assumption is frequently violated in real-world applications (*54*). In remote sensing datasets, for instance, missing values often result from the exclusion of spurious observations caused by factors such as cloud contamination in optical sensors, frozen ground conditions, or radio-frequency interference in radar systems (*55*, *56*). Missing values complicate the derivation of a general mathematical relationship between $\lambda_{\text{Var}}$ and $\lambda_{\text{AC}1}$, as the relationship becomes sensitive to the specific pattern and distribution of the gaps. To systematically assess the impact, we introduce artificial missing values into synthetic time series, allowing a controlled evaluation of their influence on CSD-based indicators.

**Fig. 2.**
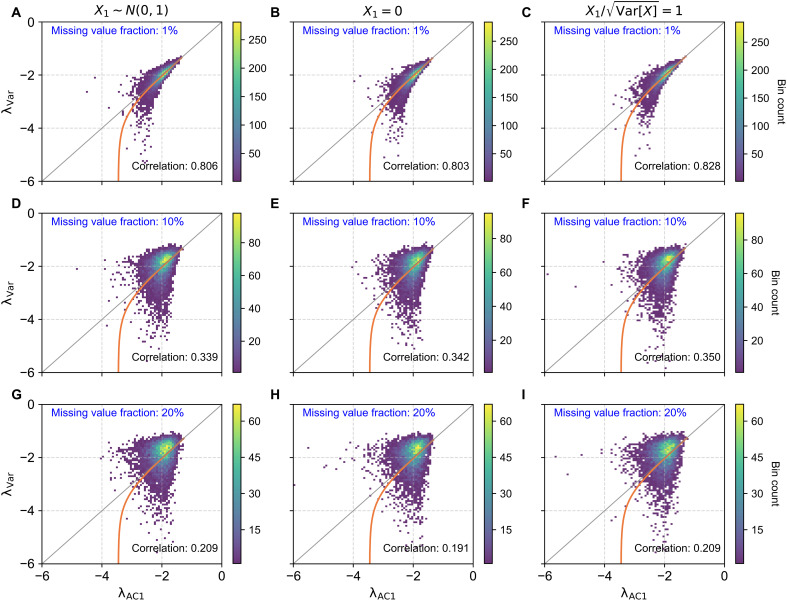
Relationship between $\lambda_{\text{Var}}$ and $\lambda_{\text{AC}1}$ under varying fractions of missing values. Each condition consists of *n* = 10,000 time series generated from an AR(1) process, each of length 1000. Missing data were introduced randomly with fractions of 1% (**A** to **C**), 10% (**D** to **F**), and 20% (**G** to **I**). The columns correspond to three distinct initial value ($X_{1}$) settings, consistent with the treatments presented in Fig. 1. The orange curve represents the universal function described by Eq. 5, which no longer serves as the lower bound when the time series contains missing values.

We generate synthetic time series (n=10,000), using an AR(1) process with parameters $\alpha =e^{-2}$ and $\sigma_{\varepsilon }=1$. Missing values are introduced by randomly removing $N_{m}$ data points from each series, where the missing value fraction is defined as $r=N_{m}/N$. Resilience indicators $\lambda_{\text{Var}}$ and $\lambda_{\text{AC}1}$ are computed using only available (nonmissing) data points (see the Supplementary Materials for the exact formulas). As illustrated in Fig. 2, the presence of missing values substantially alters the relationship between $\lambda_{\text{AC}1}$ and $\lambda_{\text{Var}}$. Even a small missing value fraction (r=1%; Fig. 2, A to C) can distort the scatter plots compared to the gap-free case (Fig. 1). As the fraction of missing data increases, the distinctions among the three initial conditions diminish, and the universal function (orange curve) no longer acts as a lower bound. These observations demonstrate that Eq. 4 does not hold in the presence of missing values. Notably, increasing missing value fraction from r=1% to r=20% results in greater dispersion in the scatter plots and a marked decrease in the correlation between the two indicators. This reduced agreement arises because missing values affect the estimation of AC1 and variance in distinct ways. Specifically, a single missing value, $X_{k}$, removes two consecutive data pairs, $\left(X_{k-1},X_{k}\right)$ and $\left(X_{k},X_{k+1}\right)$, from the AC1 regression, whereas only $X_{k}$ itself is excluded from the variance estimate. As a result, AC1 and variance are derived from different subsets of data, leading to inconsistent summation limits and a breakdown of the theoretical relationship between the two indicators. Because $\lambda_{\text{Var}}$ depends on both AC1 and variance, this discrepancy makes it inherently more sensitive to missing values than $\lambda_{\text{AC}1}$. These differential effects are further illustrated in fig. S3. Moreover, this bias cannot be resolved through simple subset adjustments (fig. S4), suggesting that robust gap-filling methods may provide the most effective means of mitigation.

### Real-world impact on global vegetation

To illustrate how missing values affect resilience estimation in real-world settings, we use global vegetation datasets derived from the MODIS as a case study. In particular, we focus on five vegetation indices: NDVI, kernel-normalized difference vegetation index (kNDVI), enhanced vegetation index (EVI), gross primary productivity (GPP), and leaf area index (LAI; see Methods). This analysis also helps to explain a previously reported phenomenon by Smith and Boers (*51*), who observed that the relationship between $\lambda_{\text{Var}}$ and $\lambda_{\text{AC}1}$ varies substantially across land-cover types. We propose that this variation is substantially affected by differences in the fraction of missing values across ecosystems.

**Fig. 3.**
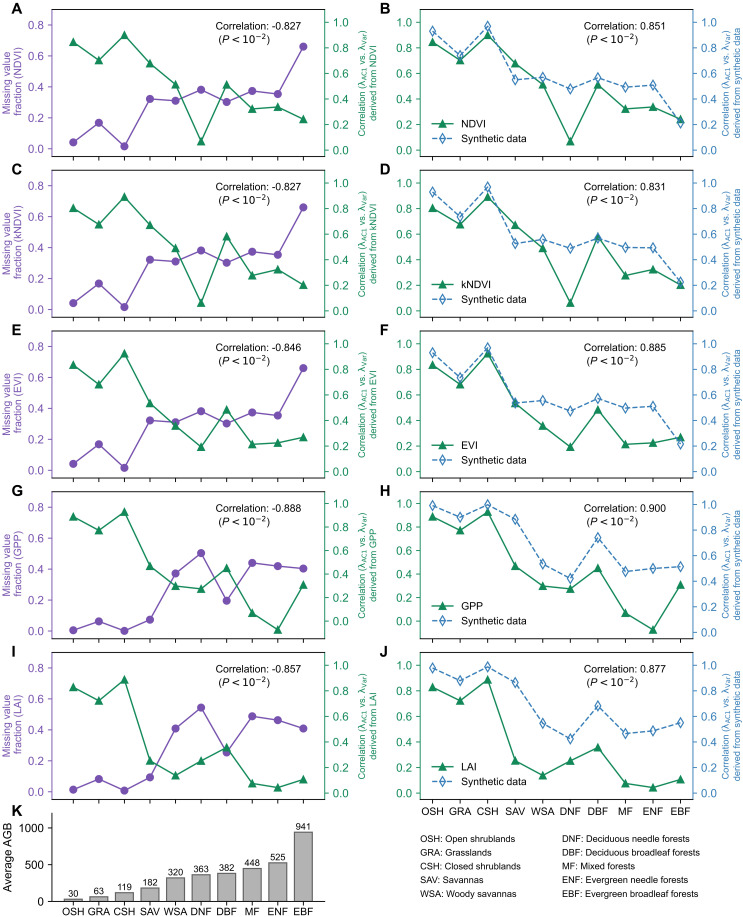
Relationship between resilience indicator consistency (correlation between $\lambda_{\text{Var}}$ and $\lambda_{\text{AC}1}$) and missing value ratios across land-cover types in MODIS and synthetic datasets. (**A**, **C**, **E**, **G**, and **I**) Resilience indicator consistency is calculated from n=10,000 points per natural land-cover type using MODIS vegetation indices at native sensor resolutions (EVI, NDVI, and kNDVI: 250 m; GPP and LAI: 500 m) (solid green lines). Median fraction of missing values across land-cover types for all MODIS vegetation indices are represented by the solid purple lines. (**B**, **D**, **F**, **H**, and **J**) Dotted blue lines represent resilience indicator consistency derived from synthetic datasets, generated using an AR(1) process that incorporates the fraction of missing values observed in the corresponding MODIS land-cover types. Pearson correlation coefficients between the solid purple and green lines, and between solid green and dotted blue lines, are indicated in each panel. (**K**) Histogram of average above-ground biomass (AGB) density across ten land-cover types. All panels share the same *x*-axis labels, representing land-cover types ordered by decreasing average AGB.

On a global scale, the MODIS data reveal a strong association between the fraction of missing values and regional biomass. As illustrated by the purple lines in Fig. 3, data from high-biomass land-cover types, such as evergreen forests, consistently exhibit a greater share of missing observations. In contrast, data from low-biomass land-cover types, such as open shrublands, experience lower fractions of missing values. This spatial correspondence is further supported by the similarity between land-cover (i.e., biomass) distributions (fig. S5) and the missing values distribution of the MODIS NDVI dataset (fig. S6). One key cause of this pattern lies in atmospheric conditions: high static stability (especially in the sinking branch of the Hadley cell in the subtropics) is associated with low cloud coverage, low precipitation, and hence low biomass, whereas tropical, high-biomass regions typically exhibit high cloud coverage (*57*, *58*). Moist and cloudy atmospheric conditions obstruct optical and thermal satellite sensors, leading to more data loss compared to arid regions.

Given that missing values weaken the agreement between $\lambda_{\text{Var}}$ and $\lambda_{\text{AC}1}$, their prevalence in high-biomass ecosystems likely contributes to the reduced indicator agreement observed in these regions (*51*). This interpretation is supported by a strong negative correlation between the missing value fraction (solid purple lines in Fig. 3) and the agreement between $\lambda_{\text{Var}}$ and $\lambda_{\text{AC}1}$ (solid green lines in Fig. 3) across various land-cover types. This pattern is robust across all five MODIS indices considered, underscoring a strong link between missing value fraction and reduced agreement between resilience indicators.

We further assess the influence of missing values on the agreement between $\lambda_{\text{Var}}$ and $\lambda_{\text{AC}1}$ in remote sensing data through a series of synthetic experiments. For each MODIS vegetation dataset, we generate a corresponding synthetic dataset using an AR(1) model with parameters $\alpha =e^{-1}$ and $\sigma_{\varepsilon }=1$ (see fig. S7 for other parameter settings). These synthetic datasets match the remote sensing datasets in both the number and length of time series (n=10,000 for each natural land-cover type, following the stratified sampling design described in the Methods). Critically, we ensure that the proportion of missing values for each land-cover type in the synthetic datasets mirrors that observed in the remote sensing data. For example, the evergreen vegetation class in the synthetic NDVI dataset maintains the same proportion of missing values as observed in the MODIS NDVI dataset for evergreen vegetation. This design guarantees that any difference observed in resilience indicators arises solely from missing value patterns, rather than from other data characteristics or underlying dynamic processes. As shown in the Fig. 3 (B, D, F, H, and J), the correlation between $\lambda_{\text{Var}}$ and $\lambda_{\text{AC}1}$ in the synthetic datasets (dotted blue lines) exhibits strong agreement with the results computed from the corresponding remote sensing datasets (solid green line). This agreement holds true for different MODIS vegetation indices with different spatial resolutions (fig. S8), which confirms that the observed divergence of the $\lambda_{\text{Var}}$-$\lambda_{\text{AC}1}$ relationship across land-cover types is primarily driven by differences in the fraction of missing values. We emphasize that we can broadly reproduce this divergence using only the missing value fraction, and ignoring underlying differences in different ecosystems (e.g., intrinsic timescales of vegetation in forest vs savannah). This implies both a very strong control on resilience estimates by missing values and a strong underlying similarity in the dynamics of different vegetated ecosystems. To ensure this result is not an artifact of the deseasoning method, we repeated the analysis using alternative methods [e.g., seasonal-trend decomposition using LOESS (STL)], which yield consistent results (fig. S9), reinforcing the robustness of this conclusion.

### Outliers introduce systematic biases in resilience assessments

Beyond missing values, our theoretical analysis in the form of Eq. 4 and Fig. 1 also suggests that outliers may introduce additional systematic biases in resilience indicators, because they can affect the variance ratio between the first data point and the rest of the data. Such biases are common across many types of empirical time series and are particularly relevant for satellite-derived products, which are often affected by spurious data points that deviate from expected annual or seasonal patterns (*59*). Here, we define outliers as data points that deviate from the mean μ by more than *k* standard deviations σ, i.e., μ±kσ. These outliers may arise from various sources, including atmospheric effects (e.g., cloud cover, aerosol interference causing over- or underestimation of vegetation greenness) (*60*), sun angle and topographic shadow effects (e.g., shadowing leading to artificially low NDVI values in mountainous terrain) (*61*), and instrument limitations (e.g., receiver sensitivity to connectivity, weather, topography, and canopy interference) (*62*), among others. Furthermore, as CSD-based analyses typically rely on anomaly time series obtained after detrending and deseasonalizing the data, inappropriate or suboptimal preprocessing methods can also introduce spurious outliers (*51*).

For time series without missing values, the relationship between $\lambda_{\text{Var}}$ and $\lambda_{\text{AC}1}$, as described by Eq. 4, is governed by the relative amplitude of the first data point, $X_{1}^{2}/\text{Var}\left[X\right]$. Consequently, the influence of outliers can be understood under two conditions. First, an outlier occurring at the first data point ($X_{1}$) directly modifies the relationship between the two indicators. Second, when outliers are present elsewhere in the time series, particularly if they are numerous or of large magnitude, they increase the overall variance, thereby diminishing the relative weight of $X_{1}^{2}/\text{Var}\left[X\right]$. In this case, the relationship between $\lambda_{\text{Var}}$ and $\lambda_{\text{AC}1}$ tends to converge toward the universal lower bound defined by Eq. 5. This behavior is confirmed numerically in fig. S10.

For time series with missing values, however, the influence of outliers on $\lambda_{\text{Var}}$ and $\lambda_{\text{AC}1}$ cannot be understood in such an algebraic way. Missing values mean that there are, in effect, several time series points at the beginning and end of the interrupted time series parts. These all play into the relation between $\lambda_{\text{Var}}$ and $\lambda_{\text{AC}1}$ in an algebraically intransparent way. We can a priori not expect any specific change in correlation between the two quantities with respect to an outlier in the first data point. Nonetheless, we can investigate these effects through synthetic time series experiments.

**Fig. 4.**
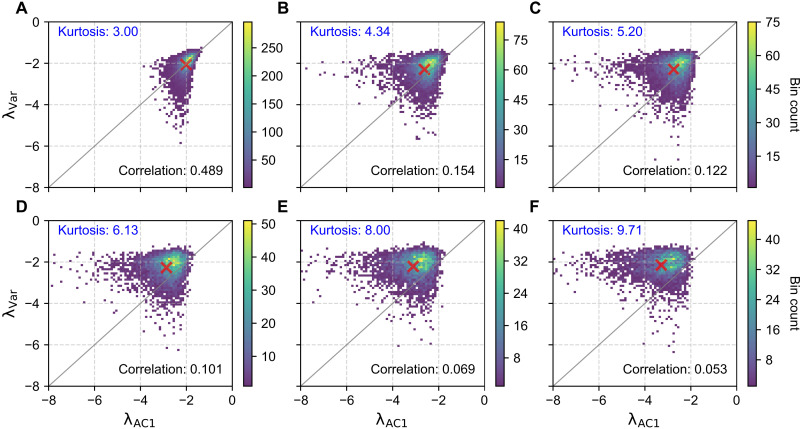
Relationship between $\lambda_{\text{Var}}$ and $\lambda_{\text{AC}1}$ under varying outlier magnitudes. Each panel shows results from n=10,000 simulated time series generated from an AR(1) process, each of length 1000. Five percent of the values are randomly designated as missing, and an additional 5% are set as outliers. (**A**) Baseline case with no outliers. (**B** to **F**) Outlier magnitude increases progressively by scaling deviations with parameter *k* (k=3.0,3.5,4.0,5.0,and6.0), resulting in increasing kurtosis across panels. Red crosses indicate the mean position of the scatter plot in each panel.

We generate time series using an AR(1) process with parameters $\alpha =e^{-2}$ and $\sigma_{\varepsilon }=1$ (see fig. S11 for other parameter settings). Five percent of the values in each series are randomly designated as missing, while additional 5% are assigned as artificial outliers. These outliers are generated by randomly sampling from the intervals [μ+kσ,μ+(k+1)σ] or [μ−(k+1)σ,μ−kσ]. The parameter *k* controls the magnitude of deviation, with larger values producing more extreme deviations from the mean. An example of artificial outliers is illustrated in fig. S12. The magnitude of outliers is quantified by computing the kurtosis of the dataset (see the Supplementary Materials), a widely adopted statistical measure for outlier detection (*63*, *64*). By varying *k*, we assess the impact of increasing outlier magnitude on the performance of resilience indicators. As shown in Fig. 4, higher outlier magnitudes, as reflected in increased kurtosis, lead to a reduced correlation between the two resilience indicators. Notably, the presence of outliers substantially alters the distributional properties of $\lambda_{\text{Var}}$ and $\lambda_{\text{AC}1}$. In the baseline case with kurtosis equal to 3, where time series are affected only by missing values and contain no outliers (Fig. 4A), the scatter plot of $\lambda_{\text{Var}}$ versus $\lambda_{\text{AC}1}$ is evenly distributed around the 1:1 line, forming what we refer to as the “balanced pattern.” In this pattern, the mean position of the scatter points (red cross) lies on the 1:1 line, indicating that the average deviation between $\lambda_{\text{Var}}$ and $\lambda_{\text{AC}1}$ across the dataset is close to zero. This suggests that, despite point-wise variability, averaging resilience indicators over the full dataset still yields a consistent estimate of system resilience. As outlier magnitude increases (Fig. 4, B to F), the distribution progressively shifts toward the upper left of the 1:1 line, forming a “biased pattern.” Here, the mean position of the scatter points moves above the 1:1 line, indicating a systematic divergence in which $\lambda_{\text{AC}1}$ is consistently lower than $\lambda_{\text{Var}}$. As a result, averaging the two resilience indicators across the dataset (e.g., the red cross in Fig. 4F) yields an inconsistent estimate of system resilience, reflecting the distortion introduced by outliers.

To investigate the mechanism behind this divergence, we separately examine the sensitivity of each indicator to outlier magnitude. We find that $\lambda_{\text{Var}}$ remains relatively stable to increasing outlier magnitude. This stability arises from its formulation in Eq. 2: although both ${\hat{\sigma }}_{\varepsilon }^{2}$ and Var[X] increase with stronger outliers, their ratio remains relatively stable (fig. S13). In contrast, $\lambda_{\text{AC}1}$ decreases as outlier magnitude increases. This decline arises because randomly placed outliers disrupt the autocorrelation structure, weakening the correlation between successive values and thus reduce AC1-based resilience estimates. As a result, outliers systematically impose a negative bias on $\lambda_{\text{AC}1}$ (a shift to the left in Fig. 4). This leads to an overestimation of resilience when relying on the AC1 indicator and, thus, potentially to late warning of forthcoming transitions.

**Fig. 5.**
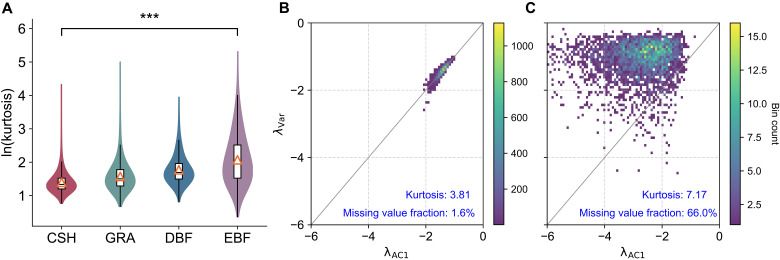
Outlier and missing value induced shifts in the relationship between resilience indicators. (**A**) Kurtosis of (deseasonalized and detrended) NDVI data. The central line denotes the median, the lower and upper hinges represent the 25th and 75th percentiles, respectively, whiskers extend to the 95% confidence intervals, and triangles indicate the mean. Significant differences in kurtosis (P<0.001) between closed shrublands and evergreen broadleaf forests, identified by ANOVA, are marked with asterisks. (**B** and **C**) Influence of missing values and outliers on the distributional properties of $\lambda_{\text{Var}}$ and $\lambda_{\text{AC}1}$. Simulated time series demonstrate a shift from a balanced pattern (B) to a biased pattern (C) as both the fraction of missing values and the magnitude of outliers increase. Outliers were introduced by designating 4% of data points as extreme values, with the outlier magnitude parameter *k* increasing from 2.67 (B) to 8.50 (C). The fraction of missing values and kurtosis values in (B) and (C) match those observed in MODIS NDVI data for shrublands (fraction of missing values = 1.6%, median kurtosis = 3.81) and evergreen broadleaf forests (fraction of missing values = 66.0%, median kurtosis = 7.17), respectively.

This transition from balanced to biased patterns is also observable in satellite-derived vegetation indices. Specifically, time series from evergreen broadleaf forests display a biased pattern, whereas those from closed shrublands exhibit a balanced pattern [see fig. S14 or figure 2 (a and b) in (*51*)]. These differences align with the significantly higher kurtosis values observed in evergreen forests compared to shrublands (Fig. 5A), suggesting that variations in outliers contribute to the shift in the distributional properties of $\lambda_{\text{Var}}$ and $\lambda_{\text{AC}1}$ between different land-cover types.

To further demonstrate that the distributional shift in resilience indicators between evergreen broadleaf forests and closed shrublands arises from data imperfections, we reproduced this phenomenon using synthetic time series by incorporating the observed differences in missing values and outliers between these two land-cover types. As shown in Fig. 5 (B and C), synthetic time series generated from an AR(1) process with parameters $\alpha =e^{-1}$ and $\sigma_{\varepsilon }=1$ exhibit distinct indicator patterns depending on the level of data imperfection. Specifically, a balanced pattern (Fig. 5B) emerges under low fractions of missing values and outlier magnitude, matching the fraction of missing values = 1.6% and median kurtosis = 3.81 observed in MODIS NDVI dataset for closed shrublands. In contrast, a biased pattern (Fig. 5C) arises under more severe data imperfections, with the fraction of missing values = 66.0% and median kurtosis = 7.17 aligned with those found in evergreen broadleaf forests. The close agreement between the synthetic (Fig. 5, B and C) and empirical results (fig. S14) confirms that heterogeneity in data quality, particularly differences in missing value proportions and outlier magnitude, plays a central role in shaping the relationship between resilience indicators across land-cover types in remote sensing observations.

## DISCUSSION

Persistent warming and anthropogenic pressures are reducing the resilience of Earth system components and especially the Earth’s ecosystems, raising the risk of abrupt regional shifts (*8*). Timely detection of resilience change is therefore essential (*9*). CSD-based indicators offer an alternative to disturbance-based measurements, enabling global resilience assessments and tracking stability changes across diverse domains (*27*, *34*, *38*, *65*). To mitigate spurious signals from individual indicators, recent studies have integrated multiple resilience indicators into composite frameworks, yielding more reliable assessments (*25*, *46*, *51*). However, the statistical dependencies between resilience indicators, and their sensitivity to common data issues remain poorly understood. To address this, we derive a general mathematical framework linking two widely used CSD-based resilience indicators, $\lambda_{\text{AC}1}$ and $\lambda_{\text{Var}}$, which allows us to relate their agreement to data quality in a way that is independent of the specific application. Using synthetic time series together with a global vegetation case study based on satellite observations, we show that missing values and outliers systematically undermine the consistency of resilience indicators, offering new guidance for robust resilience assessments in any field where CSD-based indicators are applied to empirical time series.

Our mathematical analysis reveals that, for complete time series (i.e., without missing values), the relationship between $\lambda_{\text{AC}1}$ and $\lambda_{\text{Var}}$ is strongly influenced by a functional dependence governed primarily by the amplitude of the first data point relative to the overall variance (Eq. 4). This finding underscores a previously unacknowledged sensitivity: The apparent agreement or disagreement between the two resilience indicators may arise solely from properties of the first data point in a time series rather than reflecting underlying CSD dynamics. As such, high consistency between $\lambda_{\text{AC}1}$ and $\lambda_{\text{Var}}$ should be interpreted with caution, as it does not inherently guarantee the appropriateness of the data for CSD analysis. As mentioned above, any dataset, even if not generated by an autoregressive process, can show such consistency between $\lambda_{\text{AC}1}$ and $\lambda_{\text{Var}}$. These findings challenge the assumption that such indicators provide independent confirmation of resilience changes (*51*, *53*, *66*, *67*) and underscore the importance of accounting for initial conditions when interpreting the coherence between indicators; this is particularly relevant for moving-window analyses, which are often used in assessing trends in AC1 and variance (*27*), as the first data point of each temporal window exerts an influence on the computed AC1 and variance and can hence bias inferred trends through time. Moreover, the potential interdependence between other resilience indicators, such as those based on estimating the drift function of a stochastic model (*68*), warrants further investigation.

We further assess the impact of missing values on resilience indicators. Introducing artificial missing values into synthetic time series reveals a strong decline in indicator agreement as the fraction of missing values increases (Fig. 2). Applying this framework to MODIS vegetation data, we find a robust negative correlation between missing value frequency and indicator agreement across land-cover types (Fig. 3). This relationship offers a clear explanation for the divergence in indicator agreement between high- and low-biomass areas reported by Smith and Boers (*51*). Rather than reflecting inherent ecological differences, this discrepancy appears to be primarily due to data quality issues. High-biomass ecosystems tend to experience more frequent cloud cover, resulting in higher rates of missing values in remote sensing data. Our findings demonstrate that these missing values could introduce biases in mean vegetation resilience estimates.

Alongside missing values, outliers represent another key data quality issue affecting resilience indicators. Beyond reducing consistency, outliers can distort the distributional properties of the relationship between $\lambda_{\text{AC}1}$ and $\lambda_{\text{Var}}$, as evidenced by changes in the shape of their scatter plots (Figs. 4 and 5). Notably, high-magnitude outliers systematically bias AC1-based indicators toward overestimating resilience, with these distortions particularly evident in vegetation observations. Comparing resilience indicators derived from remote sensing data reveals significant performance differences between evergreen forests (characterized by high outliers and frequent missing values) and open shrublands (where both issues are less prevalent). This suggests that AC1-based metrics may systematically overestimate vegetation resilience in evergreen-dominated ecosystems, such as tropical forests.

While our analysis focuses on the fraction of missing values and outlier magnitudes, other data characteristics may also influence resilience estimates and warrant further investigation. For instance, the distribution pattern of missing values (fig. S15) can introduce additional biases (*36*), increasing the number of outliers (in contrast to their magnitude, as shown above) can further reduce the agreement between resilience indicators (fig. S16), and different mechanisms generating outliers in real systems could lead to varying impacts. Although the relationship between $\lambda_{\text{AC}1}$ and $\lambda_{\text{Var}}$ provides valuable insights into data quality, it should not be regarded as a definitive measure of a dataset’s suitability for CSD analysis. Instead, we propose that comparing CSD-based resilience indicators with empirically derived recovery rates following large perturbations offers a more robust framework to evaluate the suitability of CSD methods (*27*). Nevertheless, both $\lambda_{\text{AC}1}$ and $\lambda_{\text{Var}}$ remain powerful tools for quantifying resilience in dynamical systems with alternative equilibria, provided their limitations are carefully considered.

Our results underscore the importance of continuous, high-quality datasets for reliable resilience assessment. Although remote sensing and other observational platforms hold great promise for global-scale monitoring, they are often limited by noise, missing data, and irregular sampling, issues that are especially pronounced in high-biomass and biodiverse regions that are vital to planetary resilience. Addressing data gaps while preserving key dynamical properties, such as AC1, remains a major challenge. Traditional gap-filling methods, including temporal resampling and linear interpolation, can introduce systematic biases in the higher-order statistics underlying resilience indicators even if the mean is unbiased (*36*, *46*, *51*). For example, gap filling by temporal resampling is sensitive to the relationship between the resampling window and the intrinsic timescale of the dynamics, potentially blending multiple lag structures. Similarly problematic, linear interpolation may impose artificially smooth dynamics, obscuring true variability. Emerging artificial intelligence–based reconstruction methods offer a promising alternative (*54*), but their ability to recover the underlying system dynamics, essential for resilience assessments, remains uncertain and deserves further exploration.

Our estimator-fidelity perspective complements recent efforts to diagnose when and why theoretical CSD-based indicators may be misleading. For instance, in Arctic sea ice models, rising AC1 has been observed without proximity to a bifurcation, as changes in effective heat capacity widen the potential well, yielding “false alarms” when AC1 is interpreted as a universal warning signal (*43*). Similarly, idealized simulations of the Atlantic Meridional Overturning Circulation have demonstrated that CSD indicators cannot readily distinguish between bistable and monostable regimes, sometimes signaling an approaching transition that does not exist (*45*). These cases highlight that a priori dynamical understanding of the studied system is essential for physical interpretation. Our contribution is orthogonal to such dynamical perspectives: Rather than verifying theoretical assumptions, we quantify how data imperfections, specifically missing values and outliers, distort indicator statistics and their mutual consistency. This statistical lens emphasizes that reliable resilience estimates require two complementary foundations: a sound dynamical framework to interpret indicator trends, and robust data preprocessing to ensure estimator fidelity and transparency.

In conclusion, this work bridges a critical gap between theoretical resilience frameworks and their empirical application, revealing the intricate interplay between data characteristics and statistical estimators. By formalizing the relationship between $\lambda_{\text{AC}1}$ and $\lambda_{\text{Var}}$ and diagnosing the impacts of missing values and outliers, we show how data quality can compromise the robustness of resilience assessments. Although vegetated ecosystems serve as a primary application in our study, the implications of our findings extend broadly to contexts where CSD-based resilience or stability indicators are inferred from time series data.

## METHODS

### Time series estimators for resilience

In the framework of local resilience, i.e., resilience against small perturbations near a stable fixed point, the fundamental quantity of interest is the linear restoring rate λ. This framework applies to the systems under consideration, which can be understood as an Ornstein-Uhlenbeck (OU) process, where the variability can be modeled as stochastic dynamics. In such systems, small deviations from equilibrium relax exponentially toward the attractor, and the dynamics can be represented as

$$dX_{t}=\lambda X_{t}dt+\sigma dW_{t}$$ (6)

where $X_{t}$ represents deviations from equilibrium, λ<0 characterizes the local stability, σ is the noise amplitude, and $W_{t}$ is a Wiener process representing Gaussian white noise. A less negative (more positive) λ corresponds to a slower recovery from perturbations and hence a loss of resilience, whereas a more negative λ indicates enhanced resilience.

To apply the theory to observational time series data, we discretized the exact OU solution over a finite sampling interval Δt, which yields

$$X_{t+\Delta t}=e^{\lambda \Delta t}X_{t}+\sigma \int_{t}^{t+\Delta t}e^{\lambda \left(t+\Delta t-s\right)}dW_{s}$$ (7)

and the stochastic integral is Gaussian with zero mean and variance given by Itô isometry

$$\text{Var}\left[\int_{t}^{t+\Delta t}e^{\lambda \left(t+\Delta t-s\right)}dW_{s}\right]=\int_{t}^{t+\Delta t}e^{2\lambda \left(t+\Delta t-s\right)}ds=\frac{1-e^{2\lambda \Delta t}}{-2\lambda }$$ (8)

Defining the discrete-time process $X_{i}≔X_{t_{0}+i\Delta t}$ and i.i.d. standard normals $\varepsilon_{i}∼N\left(0,1\right)$, we obtain the AR(1) discretization of the OU process

$$X_{i+1}=\alpha X_{i}+\sigma_{\varepsilon }\varepsilon_{i},\;\;\alpha ≔e^{\lambda \Delta t},\;\;\sigma_{\varepsilon }^{2}=\sigma^{2}\frac{1-e^{2\lambda \Delta t}}{-2\lambda }$$ (9)

Without loss of generality, we assume Δt=1 in the following for simplicity. The AR(1) coefficient reads

$$\alpha =e^{\lambda }$$ (10)

Under stationarity, the variance of the AR(1) process satisfies

$$\text{Var}\left[X\right]=\frac{\sigma_{\varepsilon }^{2}}{1-e^{2\lambda }}$$ (11)

#### Analytic relation between the two resilience indicators

We estimate the restoring rate λ from observations $X=\left\{X_{1},\ldots ,X_{N}\right\}$ using two well-accepted indicators: the autocorrelation-based indicators (*29*, *39*), built on Eq. 10, is

$$\lambda_{\text{AC}1}=\text{log}\left(\hat{\text{AC}1}\right),\;\;\hat{\text{AC}1}=\frac{\sum_{i=1}^{N-1}X_{i}X_{i+1}}{\sum_{i=1}^{N}X_{i}^{2}}$$ (12)

where $\hat{\text{AC}1}$ is the usual least squares estimator of the lag-1 autocorrelation; the variance-based indicator (*51*), built on Eq. 11, is

$$\lambda_{\text{Var}}=\frac{1}{2}\text{log}\left(1-\frac{{\hat{\sigma }}_{\varepsilon }^{2}}{\text{Var}\left[X\right]}\right),\text{Var}\left[X\right]=\frac{1}{N}\sum_{i=1}^{N}X_{i}^{2}$$ (13)

where ${\hat{\sigma }}_{\varepsilon }^{2}$ estimates the discrete noise amplitude $\sigma_{\varepsilon }^{2}$ defined in Eq. 9, as the variance of the AR(1) regression’s residuals

$${\hat{\sigma }}_{\varepsilon }^{2}=\frac{1}{N-1}\sum_{i=1}^{N-1}{\left(X_{i+1}-\hat{\text{AC}1}\cdot X_{i}\right)}^{2}$$ (14)

Intuitively, ${\hat{\sigma }}_{\varepsilon }^{2}$ quantifies the magnitude of random fluctuations that are not explained by the short-term memory captured by $\hat{\text{AC}1}$. To make explicit the dependence between $\lambda_{\text{AC}1}$ and $\lambda_{\text{Var}}$, we expand the sum of squares in Eq. 14 (see the Supplementary Materials for details)

$$\frac{{\hat{\sigma }}_{\varepsilon }^{2}}{\text{Var}\left[X\right]}=\frac{N}{N-1}\left(1-{\hat{\text{AC}1}}^{2}\right)-\frac{1}{N-1}\frac{X_{1}^{2}+{\hat{\text{AC}1}}^{2}X_{N}^{2}}{\text{Var}\left[X\right]}$$ (15)

Substituting it into the Eq. 13, we can yield the closed-form relationship reported in the main text

$$\lambda_{\text{Var}}=\frac{1}{2}\text{log}\left\{1-\frac{N}{N-1}\left[1-\text{exp}\left(2\lambda_{\text{AC}1}\right)\right]+\frac{1}{N-1}\frac{X_{1}^{2}+\text{exp}\left(2\lambda_{\text{AC}1}\right)X_{N}^{2}}{\text{Var}\left[X\right]}\right\}$$ (16)

### Satellite data

#### Vegetation indices

To assess the consistency of resilience indicators across global vegetation ecosystems, we use five vegetation indices derived from the Moderate Resolution Imaging Spectroradiometer’s (MODIS) observations in this study: (i) EVI and (ii) NDVI from MOD13Q1 (250 m spatial resolution, 16-day composites) (*69*), (iii) GPP (MOD17A2, 500 m spatial resolution, 8-day composites) (*70*), (iv) LAI (MCD15A3H, 500 m spatial resolution, 4-day composites) (*71*), and (v) kNDVI, a nonlinear extension of NDVI, which is calculated as

$$\text{kNDVI}=\text{tanh}\left({\text{NDVI}}^{2}\right)$$ (17)

Compared to conventional NDVI indices, kNDVI provides superior handling of nonlinearity, enhanced noise resistance, and improved temporal-spatial stability (*72*). All vegetation datasets are accessible via Google Earth Engine (*73*), and only data points flagged as “highest quality” are used in our analysis. To maintain alignment with (*51*), we set the study period to 2000 to 2022 (2002 to 2022 for LAI).

#### Land-cover data

We use MODIS land-cover data (*74*) (MCD12Q1, 500 m spatial resolution, 2001 to 2021, annual) to subdivide results by land-cover type and mask out nonvegetated areas (e.g., water bodies and urban regions), based on Land Cover Type 1. In addition, to minimize the influence of anthropogenic activity and ecosystem transitions on our results, we exclude any pixels that experienced a land-cover change (e.g., from forest to agriculture or agriculture to forest) at any point during 2001 to 2021. The land-cover data were resampled to a 250 m resolution to match the vegetation datasets using nearest-neighbor resampling.

We ensure equal representation of all land-cover types when comparing the consistency of resilience indicators by using a stratified random sample of 100,000 locations, evenly distributed across the 10 relevant natural land-cover types based on the classification of International Geosphere-Biosphere Programme type 1 (*75*). The spatial distribution of one realization of the stratified random sample is shown in fig. S5. We also used a global above-ground biomass density estimate from the 2010 composite (*76*) to assess the relationship between the consistency of resilience indicators and biomass across land cover types.

#### Detrending and deseasoning

All CSD-based resilience indicators rely on perturbations of the state variable around its equilibrium. Therefore, the analyzed time series must be approximately stationary, requiring careful removal of long-term trends and seasonal signals (*2*). To achieve this, we apply a rolling mean detrending method, followed by the removal of a third-order harmonic function fitted to the data for deseasoning, a technique shown to be particularly effective for processing remote sensing vegetation data (*51*). As an alternative detrending and deseasoning approach, we also apply the widely used STL (*77*) to cross-check our results.

## Supplementary Materials

### This PDF file includes:

Mathematical Details Figs. S1 to S16
