# Supplementary material for: Data gaps and outliers distort critical-slowing-down-based resilience indicators
Source: Sci Adv. 2026 Mar 13;12(11):eaee1916. doi: 10.1126/sciadv.aee1916 (PMC12985669; doi:10.1126/sciadv.aee1916)
Supplement: Supplementary file 1 — Mathematical Details Figs. S1 to S16 [file sciadv.aee1916_sm.pdf]

Science Advances
AAAS

Supplementary Materials for

Data gaps and outliers distort critical-slowing-down-based resilience indicators

Teng Liu *et al.*

Corresponding author: Teng Liu, teng.liu@tum.de; Andreas Morr, andreas.morr@tum.de

Sci. Adv. **12**, eaee1916 (2026)
DOI: 10.1126/sciadv.aee1916

This PDF file includes:

Mathematical Details
Figs. S1 to S16

Mathematical Details

We analytically derive a relationship between the two resilience indicators from the main text. These are defined as

$$\lambda_{\mathrm{AC1}} = \log\left(\widehat{\mathrm{AC1}}\right), \tag{S1}$$

and

$$\lambda_{\mathrm{Var}} = \frac{1}{2}\log\left(1 - \frac{\widehat{\sigma_\varepsilon}^2}{\mathrm{Var}[X]}\right). \tag{S2}$$

These formulas in turn build on three time series estimators. Lag-1 autocorrelation is commonly estimated as

$$\widehat{\mathrm{AC1}} := \frac{\sum_{i=1}^{N-1} X_i X_{i+1}}{\sum_{i=1}^{N} X_i^2}. \tag{S3}$$

This is simultaneously the least-squares estimator of the linear regression problem for the coefficients of an AR(1) model. In this framework, the noise amplitude σ_ε is estimated by gauging the residuals:

$$\widehat{\sigma_\varepsilon}^2 := \frac{1}{N-1}\sum_{i=1}^{N-1}\left(X_{i+1} - \widehat{\mathrm{AC1}}\cdot X_i\right)^2 \tag{S4}$$

Finally, we may estimate time series variance via

$$\mathrm{Var}[X] := \frac{1}{N}\sum_{i=1}^{N} X_i^2. \tag{S5}$$

Now notice that

$$\widehat{\sigma_\varepsilon}^2 = \frac{1}{N-1}\sum_{i=1}^{N-1}(X_{i+1} - \widehat{\mathrm{AC1}}\cdot X_i)^2 \tag{S6}$$

$$= \frac{1}{N-1}\sum_{i=1}^{N-1}(X_{i+1}^2 + \widehat{\mathrm{AC1}}^2 X_i^2 - 2\widehat{\mathrm{AC1}}X_i X_{i+1}) \tag{S7}$$

$$= \frac{1}{N-1}\left(N\mathrm{Var}[X] - X_1^2 + \widehat{\mathrm{AC1}}^2(N\mathrm{Var}[X] - X_N^2) - 2\widehat{\mathrm{AC1}}N\mathrm{Var}[X]\widehat{\mathrm{AC1}}\right) \tag{S8}$$

and therefore

$$\frac{\widehat{\sigma_\varepsilon}^2}{\mathrm{Var}[X]} = \frac{N}{N-1}(1 - \widehat{\mathrm{AC1}}^2) - \frac{1}{N-1}\frac{X_1^2 + \widehat{\mathrm{AC1}}^2 X_N^2}{\mathrm{Var}[X]}. \tag{S9}$$

Inserting this into Eq. S2 and using $\widehat{\mathrm{AC1}}^2 = \exp(2\lambda_{\mathrm{AC1}})$ yields the desired expression

$$\lambda_{\mathrm{Var}} = \frac{1}{2}\log\left(1 - \frac{N}{N-1}\left(1 - \exp(2\lambda_{\mathrm{AC1}})\right) + \frac{1}{N-1}\frac{X_1^2 + \exp(2\lambda_{\mathrm{AC1}})\,X_N^2}{\mathrm{Var}[X]}\right). \tag{S10}$$

The observed mean value of λ_{AC1} across most cases analyzed in this study is sufficiently negative such that $\exp(2\lambda_{\mathrm{AC1}})$ approaches a negligible magnitude (e.g., when $\lambda_{\mathrm{AC1}} < -2$, $\exp(2\lambda_{\mathrm{AC1}}) < 2 \times 10^{-2}$). As a result, the contribution of X_N^2 becomes vanishingly small relative to X_1^2, allowing the analytical framework to be simplified without a significant loss of precision.

In this study, we computed resilience indicators from time series data that contained missing values. Missing values (NaN) were appropriately handled throughout the computation process using NumPy's NaN-aware functions (*np.nanmean*, *np.nanvar*, *np.nanstd*), which automatically exclude missing values from calculations.

The kurtosis in this study is calculated as $K = \mu_4/\sigma^4$, where μ_4 is the fourth moment about the mean value and σ is the standard deviation of the distribution.

Supplementary Figures

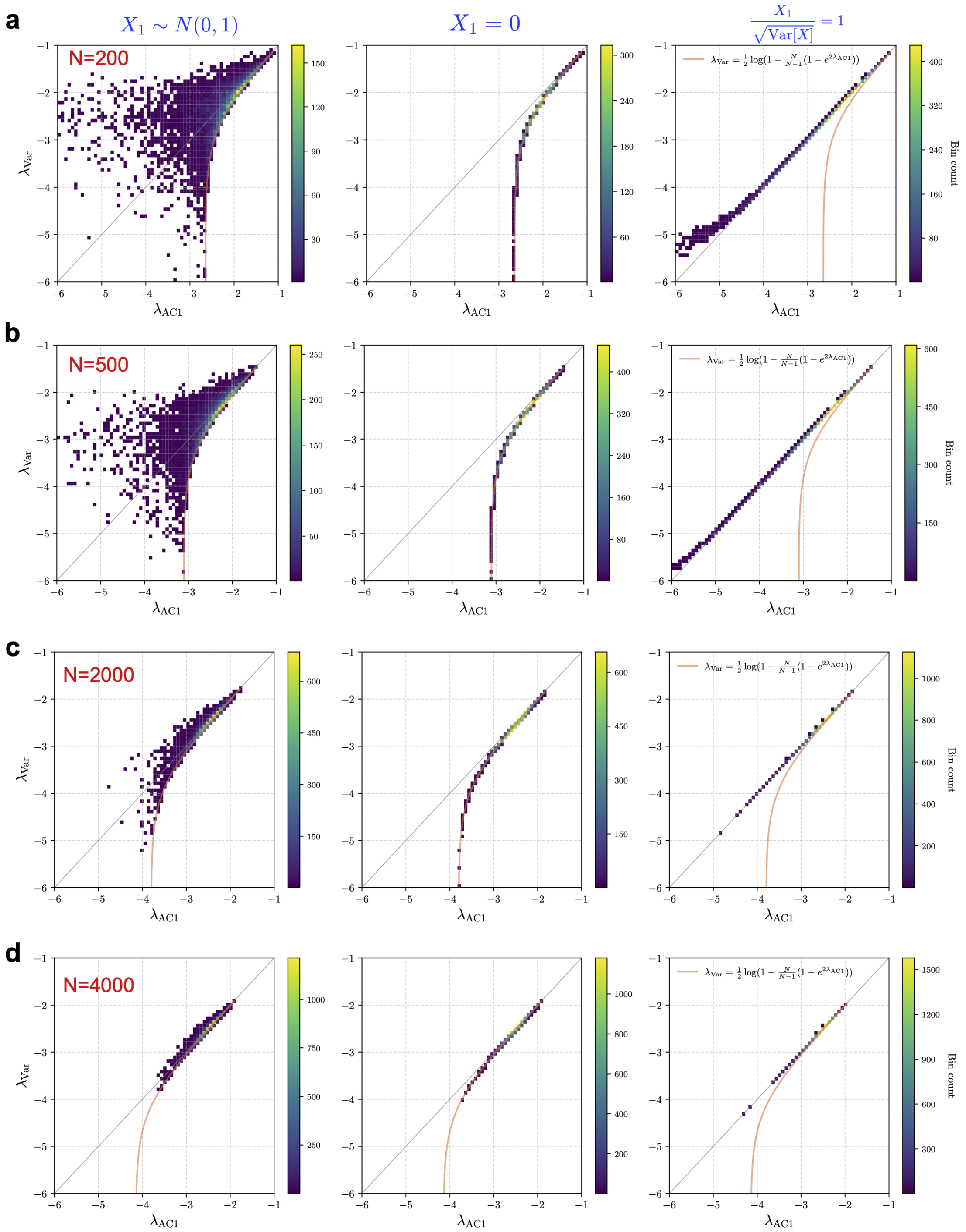

Figure S1: **Relationship between resilience indicators λ_{Var} and λ_{AC1} for different time series lengths.** (**a-d**) Same as Fig. 1 in the main text, but for time series of varying length: N = 200 (**a**), N = 500 (**b**), N = 2000 (**c**), and N = 4000 (**d**). Shorter time series exhibit a stronger dependence on the first data point, while increasing record length reduces the influence of X_1, leading to progressively tighter alignment between λ_{Var} and λ_{AC1}, consistent with Eq. S10.

Figure 1 in the main text illustrates the relationship described by Eq. S10 using time series generated from an AR(1) model. Notably, this relationship is not limited to a specific dynamical model but extends to a broader class of stochastic systems. To test the generality of our findings, we analyzed simulated time series generated from white noise (i.e., temporally uncorrelated samples drawn from a normal distribution). As shown in Figure S2, the resulting pattern closely mirrors that observed for the AR(1) process, supporting the robustness of the relationship across different types of stochastic dynamics.

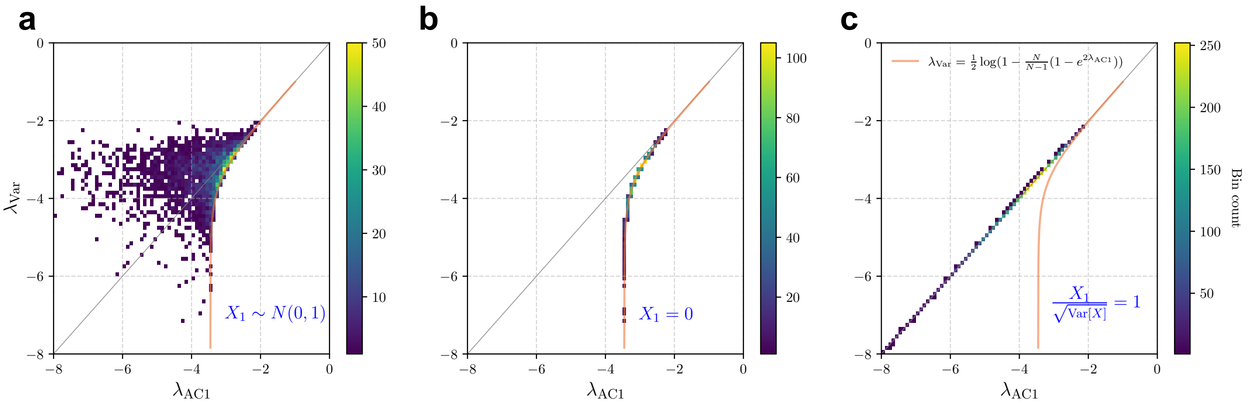

Figure S2: **Relationship between λ_{Var} and λ_{AC1} under different initial conditions.** Each condition consists of $n = 10{,}000$ time series sampled from a normal distribution $N(0,1)$, each of length 1,000. Three scenarios are considered: (**a**) The first data point is unaltered, i.e., $X_1 \sim N(0,1)$; (**b**) the first data point is artificially set as $X_1^2/\mathrm{Var}[X] = 0$; and (**c**) the first data point is artificially set to satisfy $X_1/\sqrt{\mathrm{Var}[X]} = 1$. The orange curve represents the lower bound of the distribution, described by the universal function $\lambda_{\mathrm{Var}} = \frac{1}{2}\log(1 - \frac{N}{N-1}(1 - \exp(2\lambda_{\mathrm{AC1}}))$.

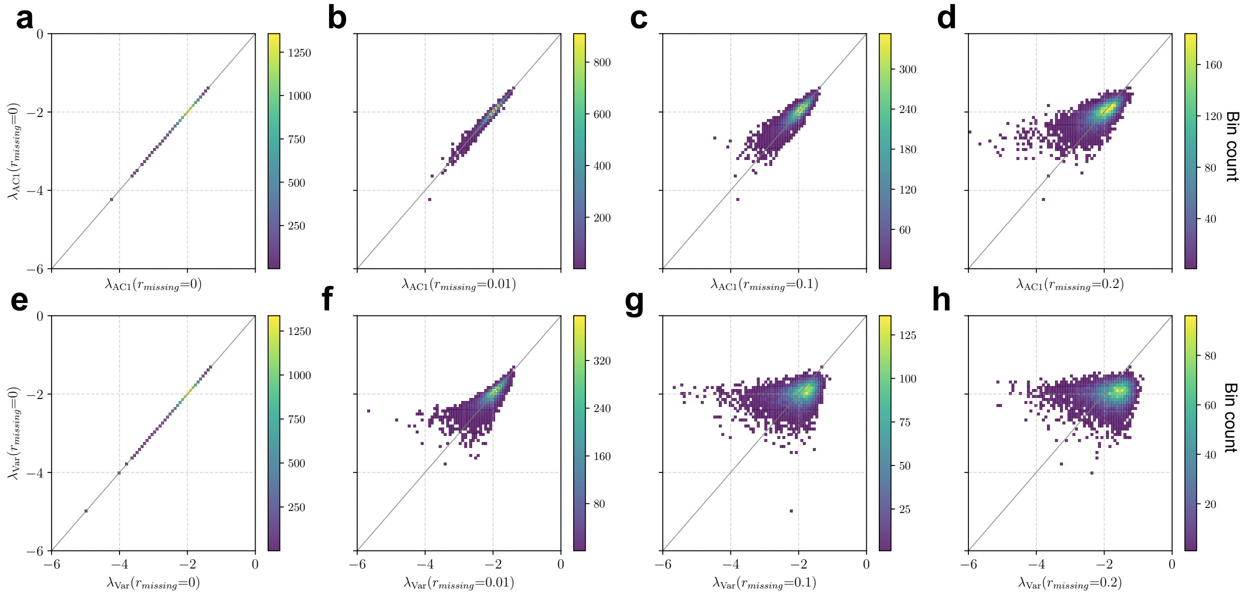

Figure S3: **Comparative sensitivity of λ_{AC1} and λ_{Var} to the missing value fraction $r_{missing}$. a-d**, λ_{AC1} values under a reference gap-free plotted against corresponding values for progressively increasing fraction of missing values ($r_{missing}$=0, 0.01, 0.1, 0.2). **e-h**, λ_{Var} under a reference gap-free plotted against corresponding values for progressively increasing fraction of missing values. The deviation from the diagonal identity line demonstrates that λ_{Var} exhibits greater sensitivity to missing values than λ_{AC1} at low missing values fraction.

We tested three simple subset adjustments to address missing values. For each missing value, X_k, we applied: (1) a forward-drop, removing X_{k+1} from the λ_{Var} calculation; (2) backward-drop, removing X_{k-1}; (3) symmetric-drop, removing both X_{k-1} and X_{k+1}. As shown in Fig. S4, none of these adjustments restored the lower-bound relationship or significantly increased the correlation.

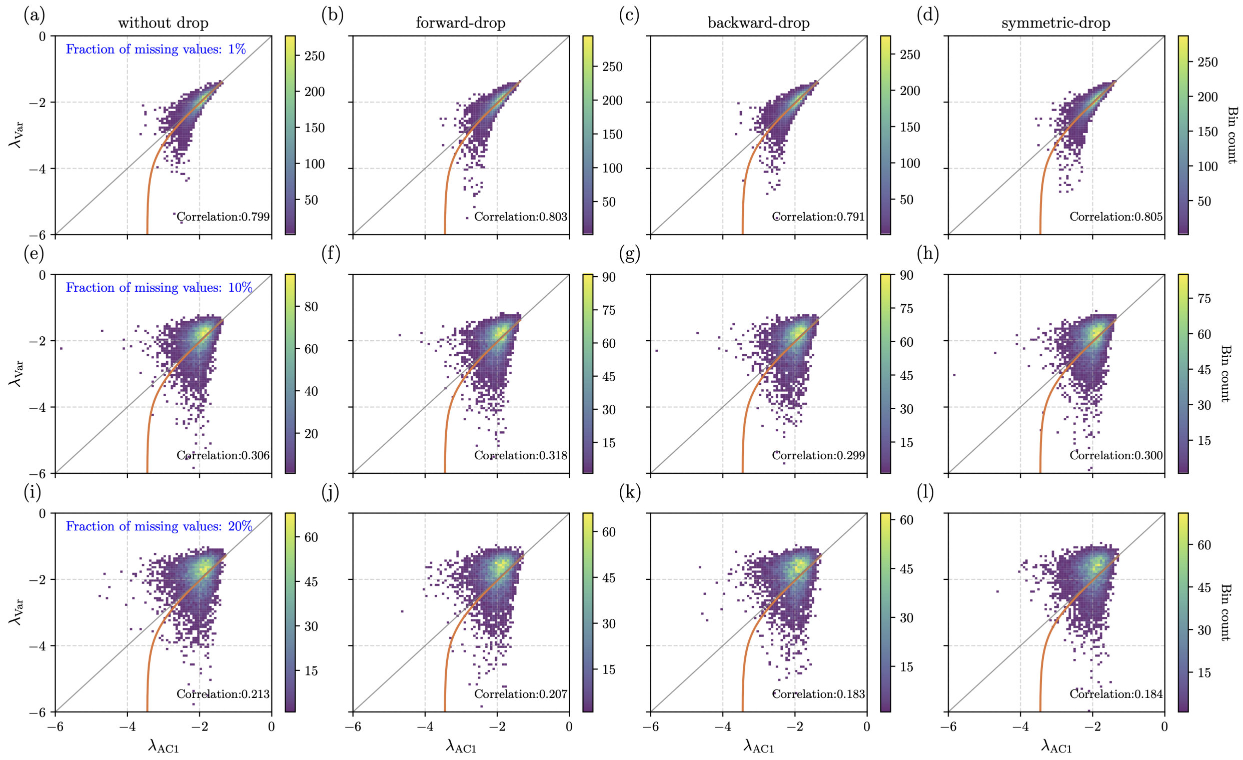

Figure S4: **Effect of alternative variance corrections on the relationship between λ_{Var} and λ_{AC1}.** Panels (**a**,**e**,**i**) correspond to (**a**,**d**,**g**) in Fig. 2 of the main text. For each missing value X_k, three gap-handling variants were tested in the variance-based indicator calculation: (**b**,**f**,**j**) forward-drop, removing X_{k+1}; (**c**,**g**,**k**) backward-drop, removing X_{k-1}; and (**d**,**h**,**l**) symmetric-drop, removing both X_{k-1} and X_{k+1}.

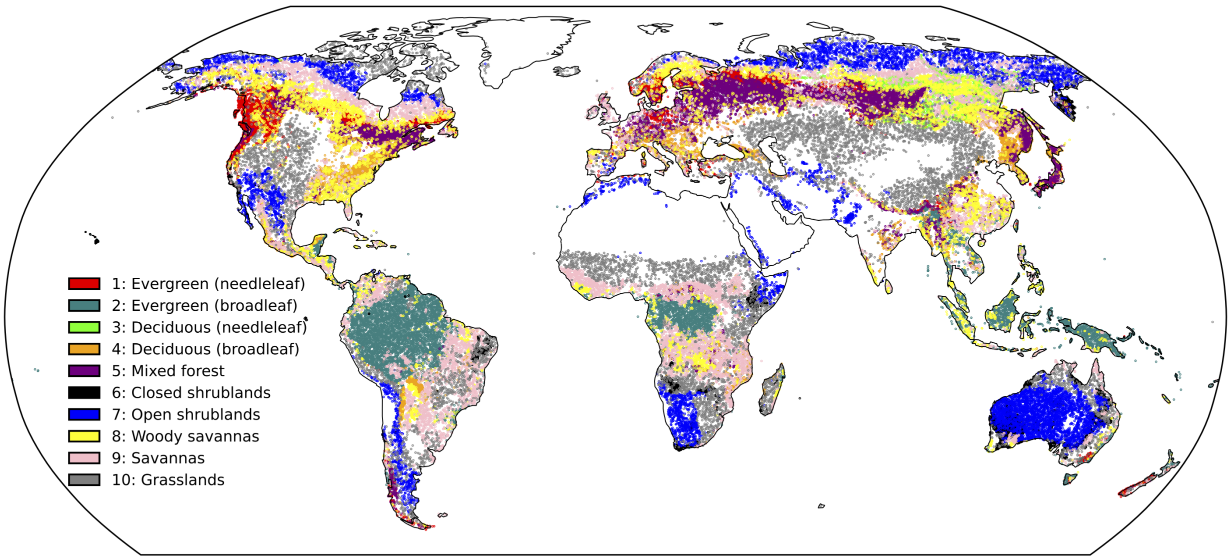

Figure S5: **Spatial distribution of land-cover type in the MODIS NDVI dataset, based on a stratified random sample of 100,000 locations (10,000 per land-cover type) across 10 natural land-cover types.**

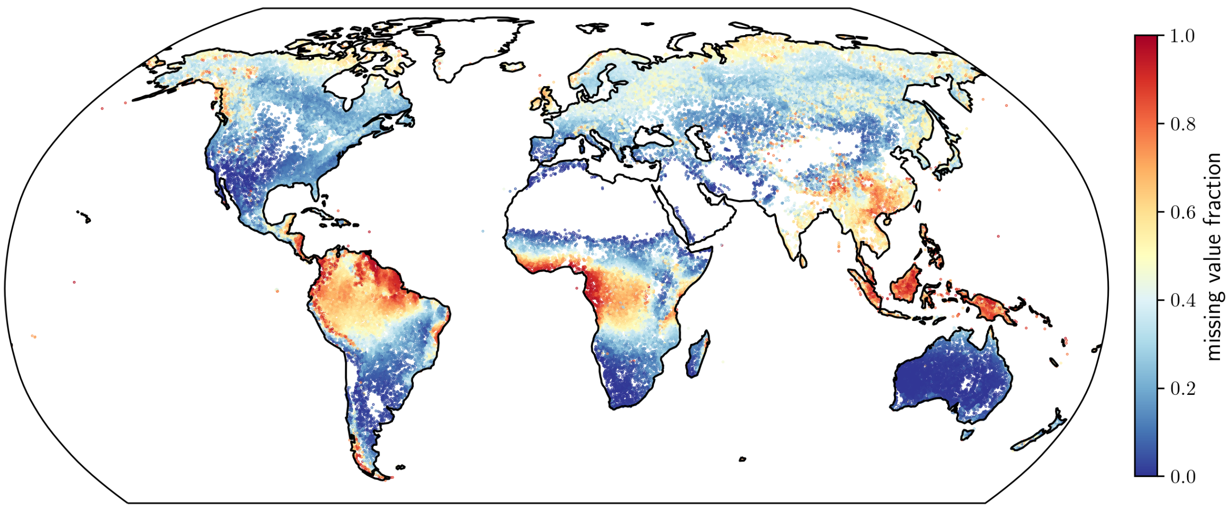

Figure S6: **Spatial distribution of missing values in the MODIS NDVI dataset, based on a stratified random sample of 100,000 locations (10,000 per land-cover type) across 10 natural land-cover types.**

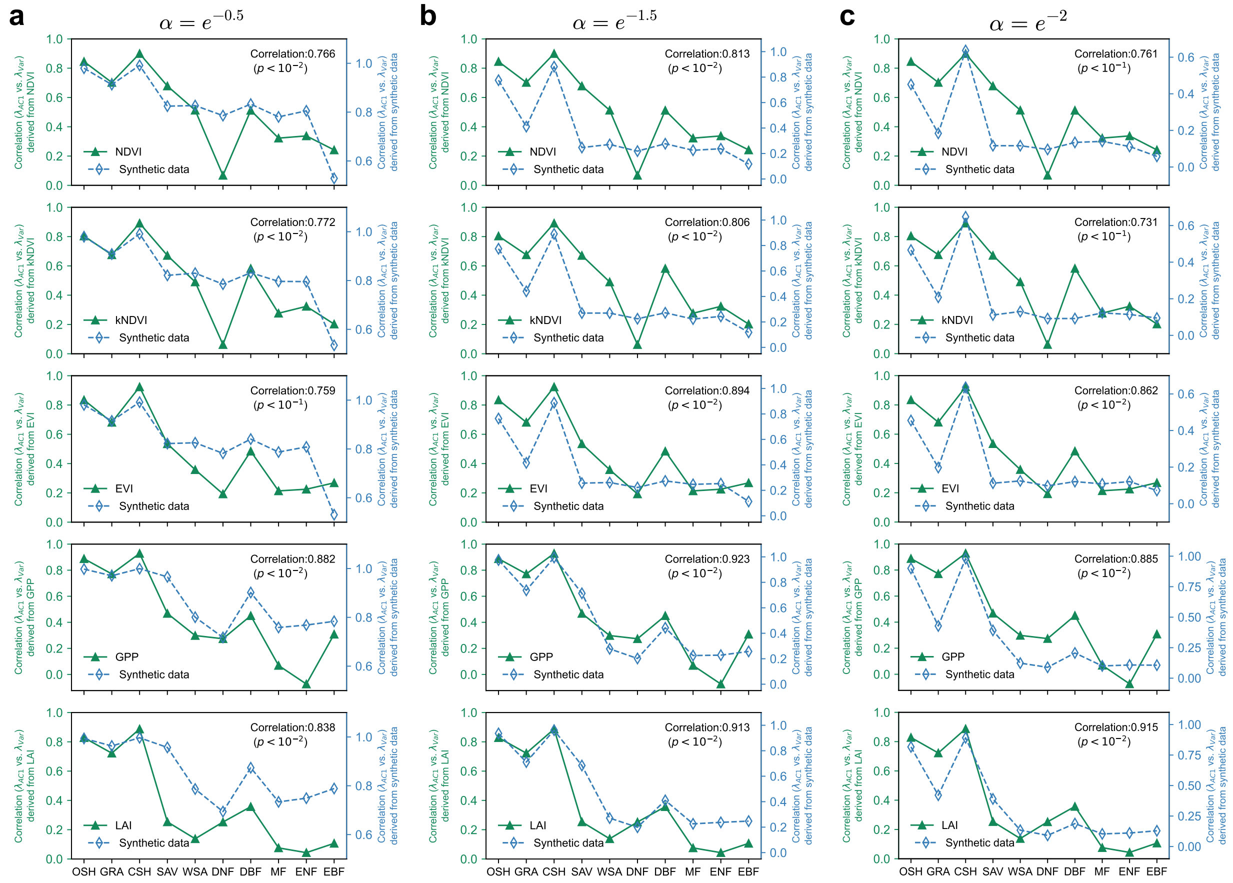

Figure S7: **Relationship between resilience indicator consistency (λ_{Var} versus λ_{AC1}) and missing value fractions across land-cover types in MODIS datasets.** This analysis follows the same approach as Fig. 3 but uses different parameter settings (α) for the synthetic dataset: $e^{-0.5}$ for **a**, $e^{-1.5}$ for **b**, and e^{-2} for **c**. The results show consistent agreement between estimates derived from remote sensing datasets (solid green lines) and those from synthetic datasets (dotted blue lines).

The robustness of the relationship between resilience indicator agreement and missing value fraction is further supported by analyses using MODIS datasets at different spatial resolutions. We derived products at 1 km resolution from the native MODIS sensor resolutions (250m for NDVI, kNDVI, and EVI; 500m for GPP and LAI) through spatial averaging implemented via Google Earth Engine. As shown in Figure S8, the results at 1 km resolution are consistent with those presented in Figure 3 of the main text, confirming that the observed patterns are not sensitive to spatial resolution.

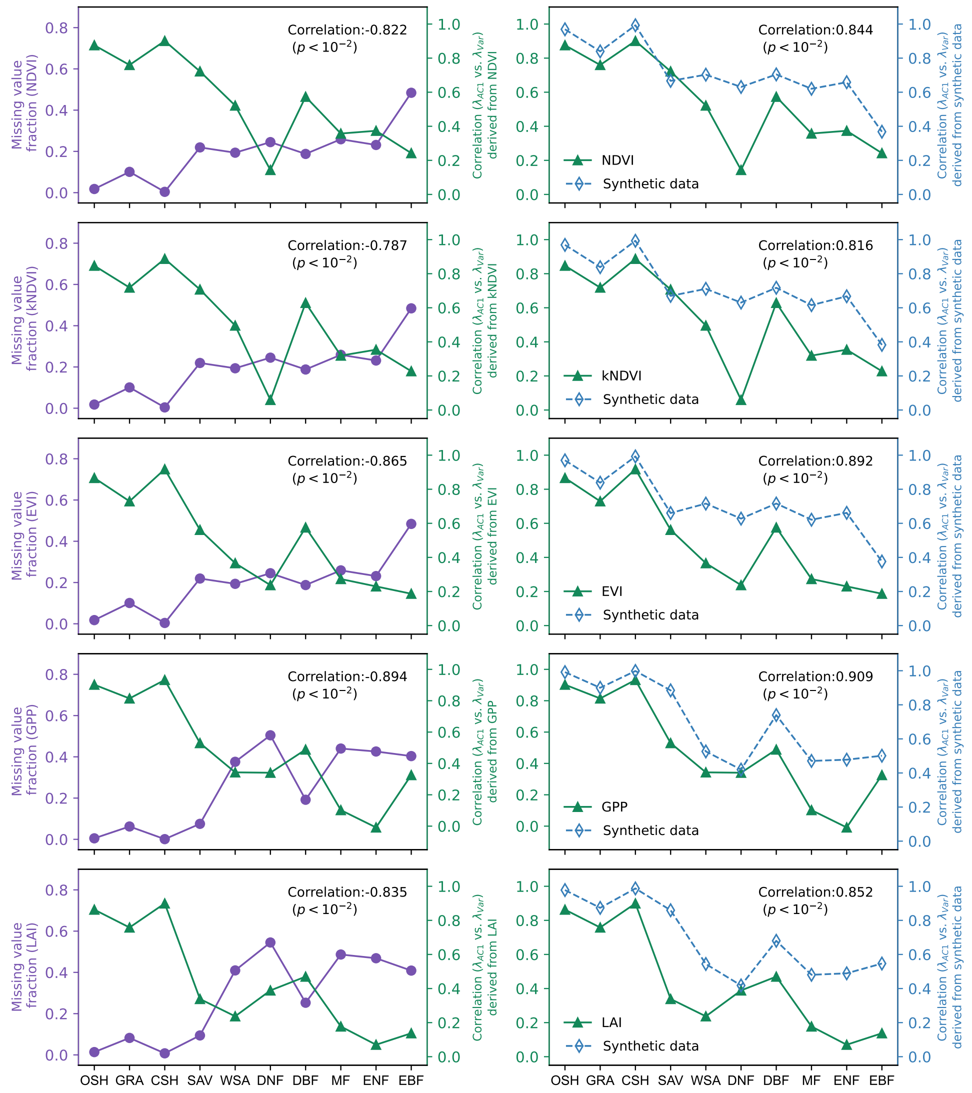

Figure S8: **Relationship between resilience indicator consistency (λ_{Var} versus λ_{AC1}) and missing value fractions across land-cover types in MODIS datasets.** This analysis follows the same approach as Fig. 3 but is based on MODIS datasets with a 1 km spatial resolution.

To assess the robustness of our findings, we implemented an alternative preprocessing approach using the widely adopted seasonal-trend decomposition based on Loess (STL) for detrending and deseasonalization (77). The resulting patterns, depicted in Figure S9, are consistent with those presented in Figure 3 of the main text, reinforcing that the observed relationship between indicator agreement and missing value fraction is independent of the specific time series decomposition technique used.

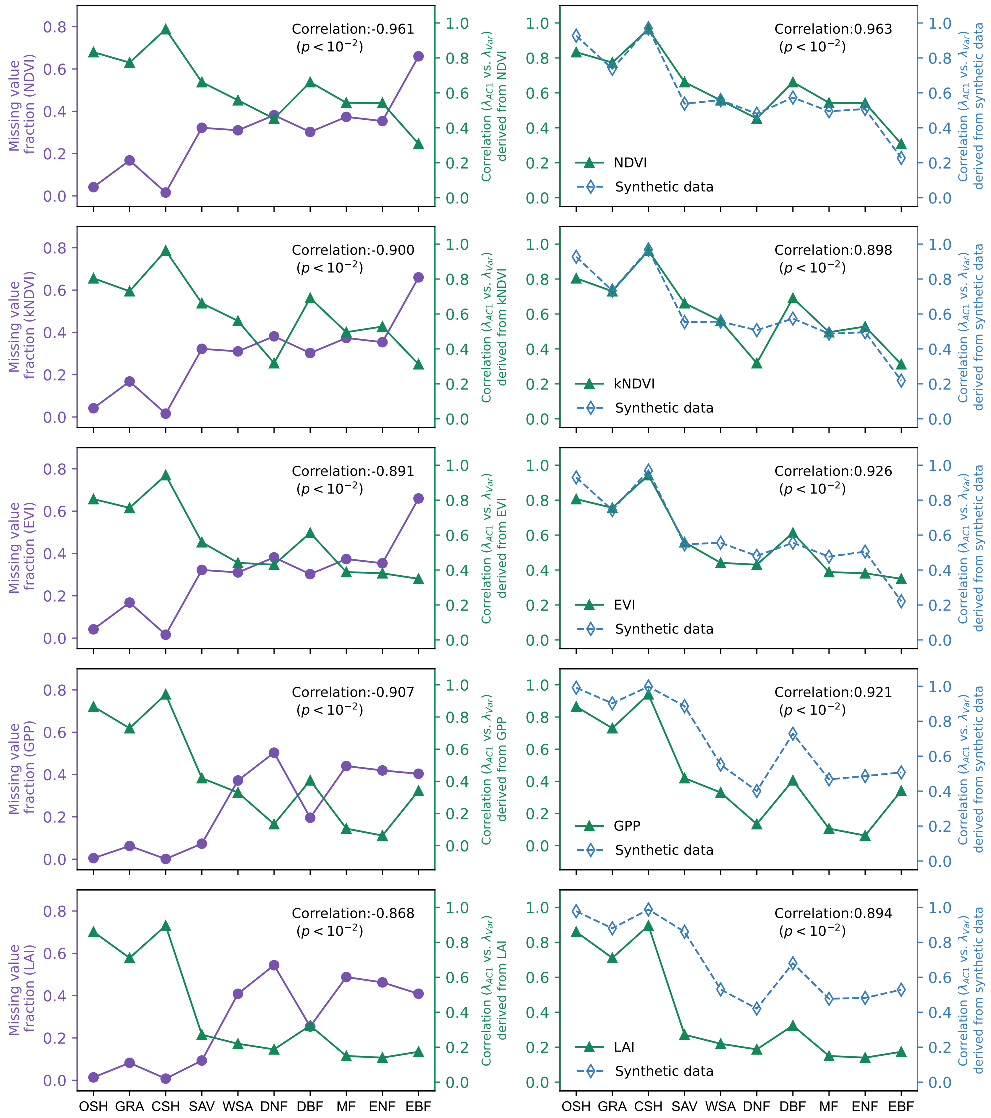

Figure S9: **Relationship between resilience indicator consistency (λ_{Var} versus λ_{AC1}) and missing value fractions across land-cover types in MODIS datasets.** This analysis follows the same approach as Fig. 3 but applies STL for detrending and deseasoning.

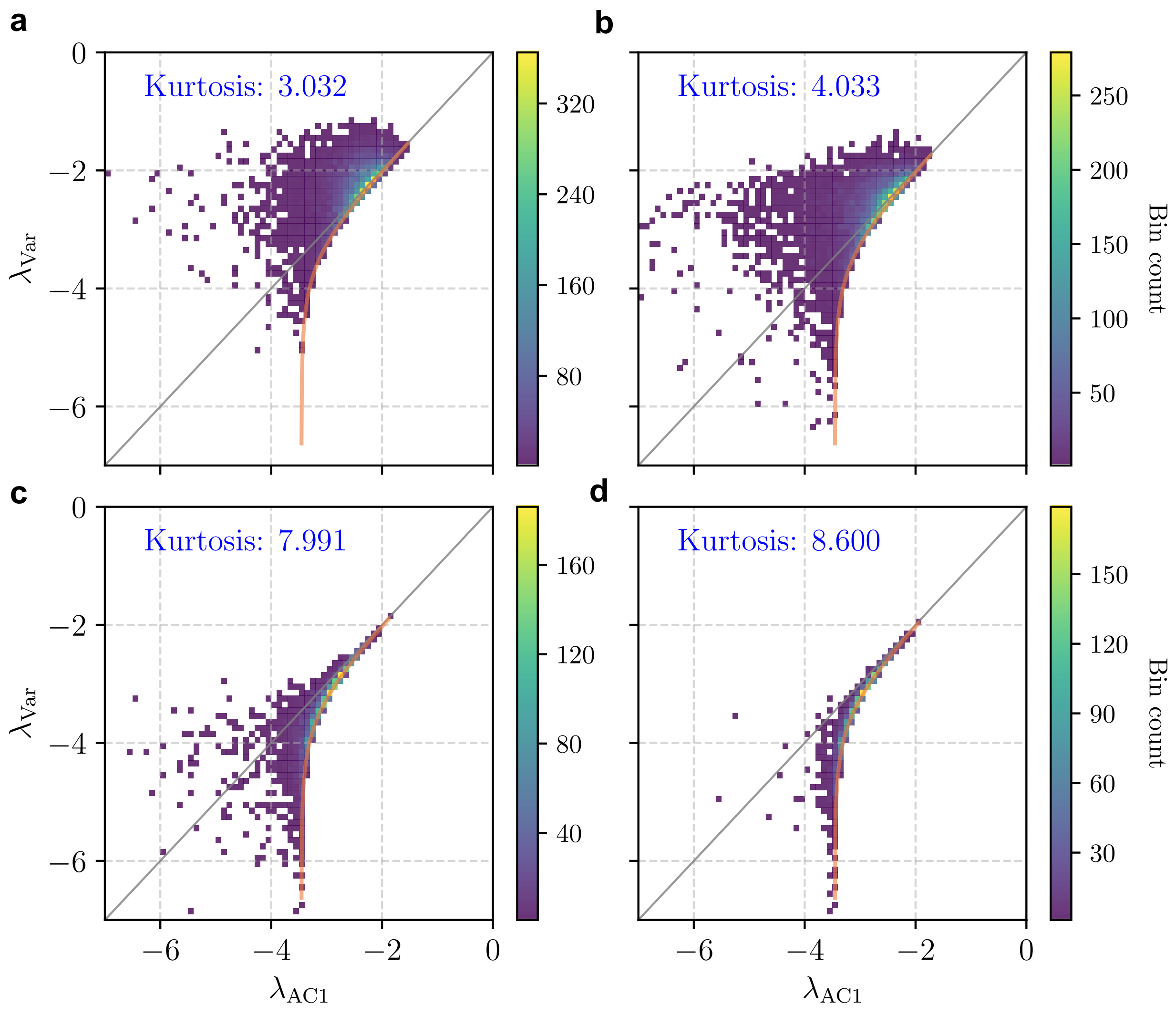

Figure S10: **Influence of outliers on the relationship between λ_{Var} and λ_{AC1} in time series without missing values.** Each panel represents results from n = 10,000 time series generated from an AR(1) process, each of length 1,000. In each condition, 10% of the values, excluding the first data point, are randomly designated as outliers. The magnitude of these outliers is progressively increased across panels **a–d**, resulting in increasing kurtosis. By avoiding changes to the initial value, we isolate the effect of outliers on overall variance. As outlier magnitude grows, the elevated variance reduces the relative contribution of the first data point ($X_1^2/\text{Var}[X]$), causing the relationship between λ_{Var} and λ_{AC1} to converge toward the universal lower bound described (orange curve).

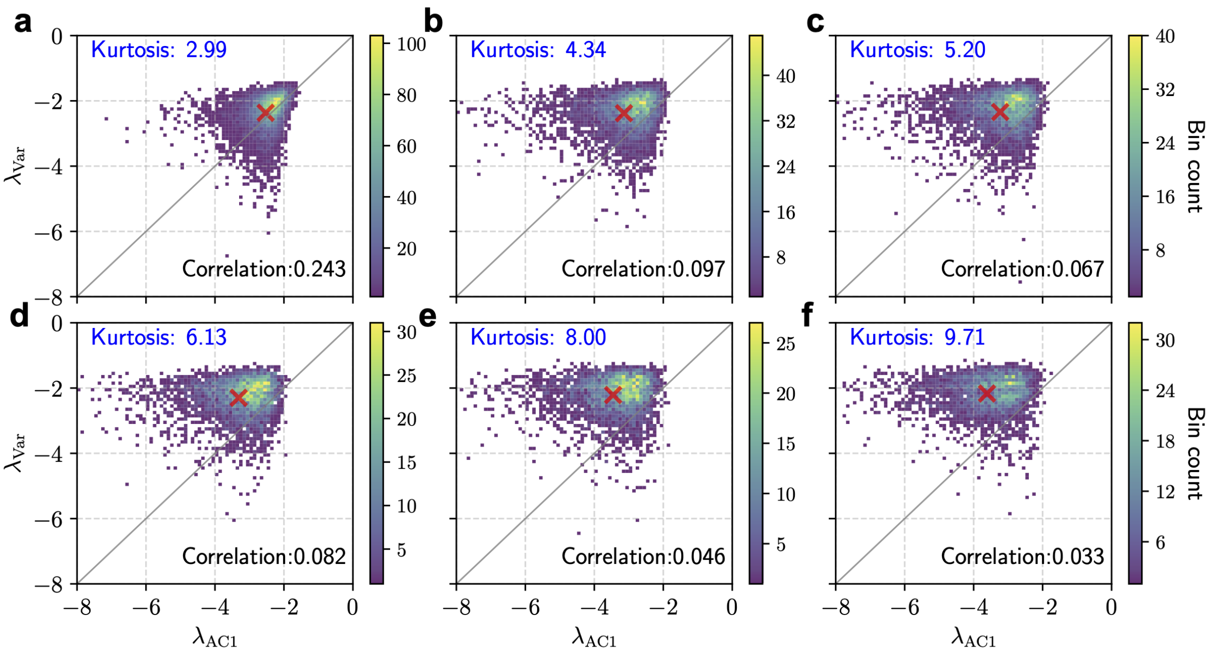

Figure S11: **Relationship between λ_{Var} and λ_{AC1} under varying outlier magnitudes.** This analysis follows the same approach as Fig. 4 but is based on the simulated time series generated from an AR(1) process with $\alpha = e^{-2.5}$. The consistent pattern between this figure and Fig. 4 indicates that the influence of outliers is unrelated to the specific parameter setting.

In the main text, we generate time series with varying outlier magnitudes to assess the impact of outliers on resilience indicators. To introduce artificial outliers, 5% of the data points are randomly replaced with values sampled from the intervals $[\mu+k\sigma, \mu+(k+1)\sigma]$ or $[\mu-(k+1)\sigma, \mu-k\sigma]$, where μ and σ denote the mean and standard deviation of the corresponding time series. The parameter k controls the magnitude of the outliers and is directly related to the resulting kurtosis, which serves as a proxy for outlier severity.

Figure S12 illustrates an example of a time series containing artificial outliers. This series was generated from an AR(1) process with parameters $\alpha = e^{-2}$, $\sigma_\varepsilon = 1$, and $k = 4$.

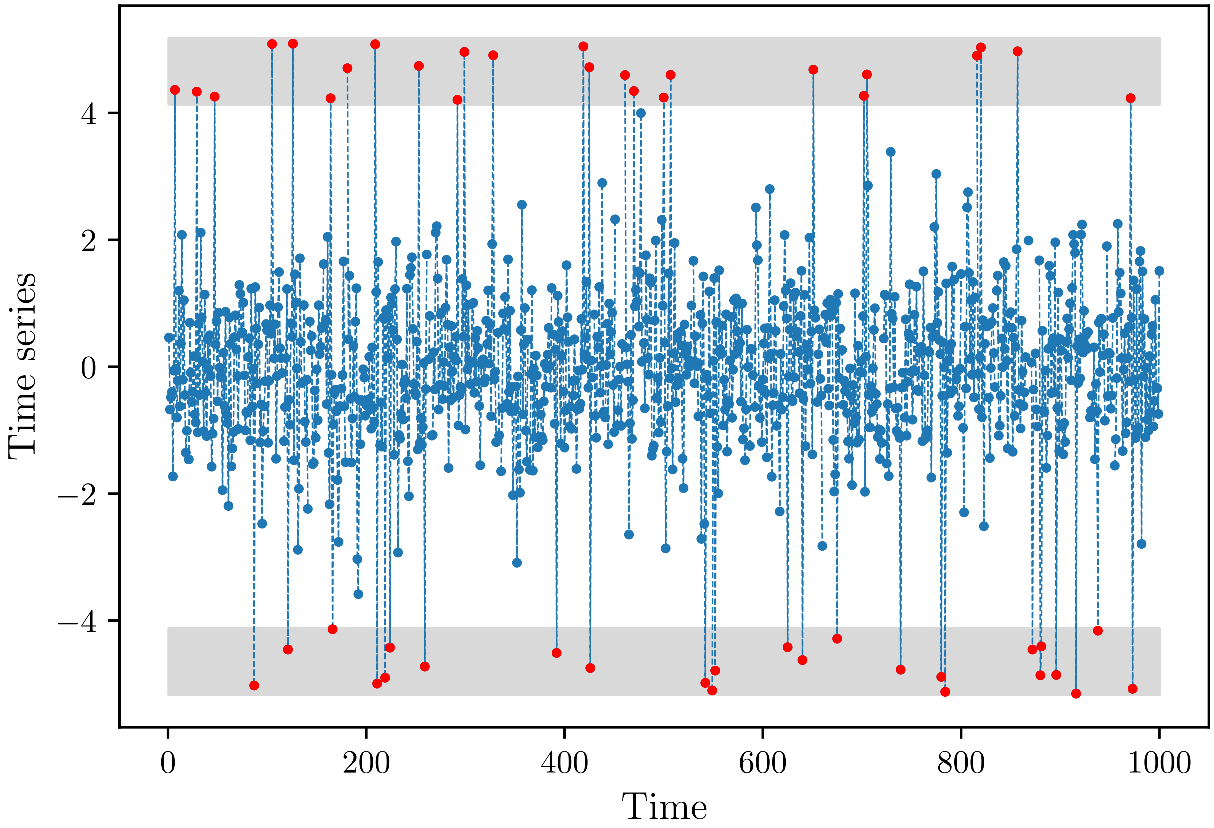

Figure S12: **Example of a time series containing artificially introduced outliers.** Red points indicate the artificial outliers, and the grey shaded areas represent the intervals $[\mu+k\sigma, \mu+(k+1)\sigma]$ and $[\mu-(k+1)\sigma, \mu-k\sigma]$, where μ and σ denote the mean and standard deviation of the time series, respectively. $k = 4$ for this example.

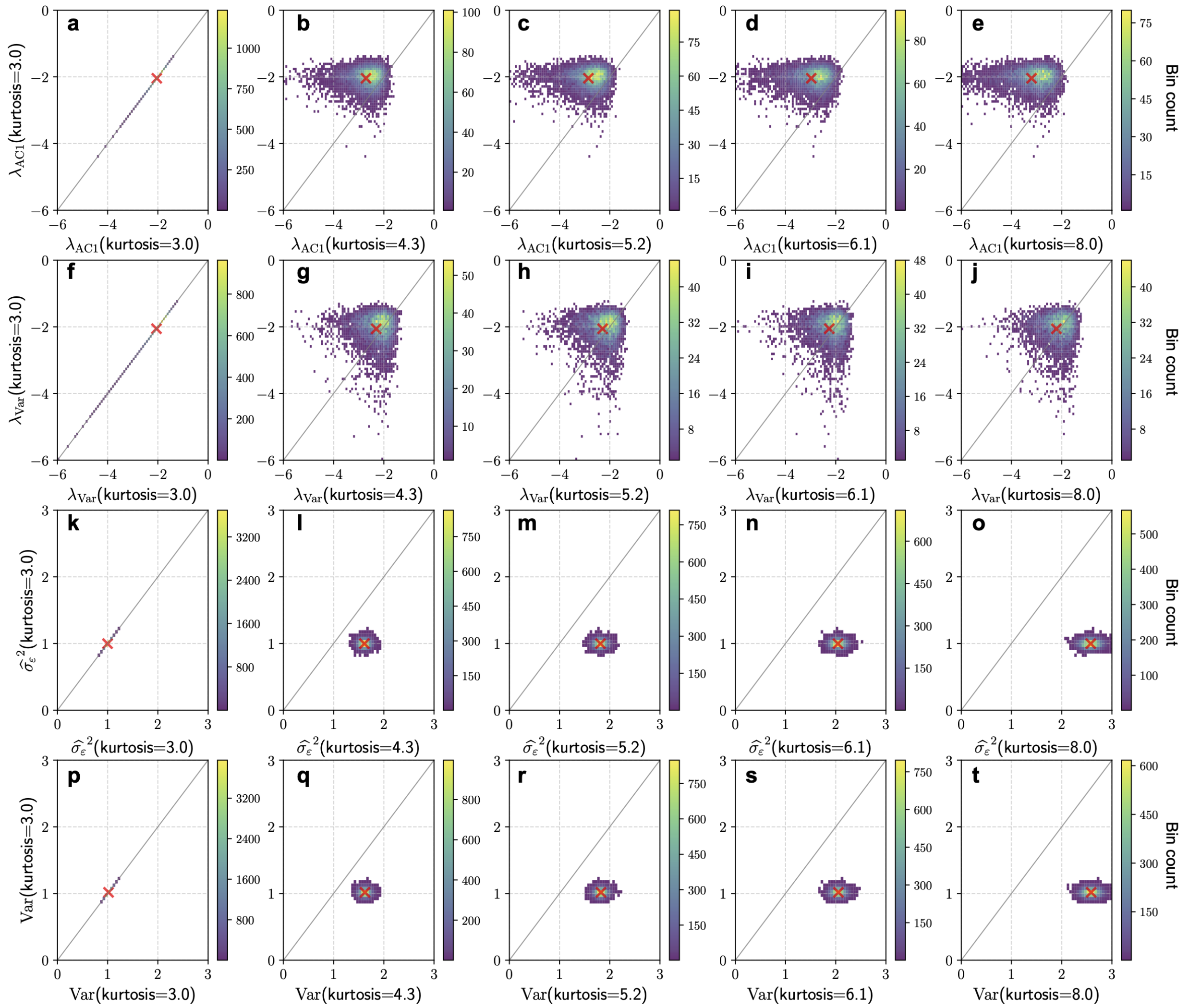

Figure S13: **Comparative sensitivity of λ_{AC1} and λ_{Var} to outlier magnitude. a-e**, λ_{AC1} values under a baseline case with no outliers plotted against corresponding values for progressively increasing kurtosis ($k = 3.0, 3.5, 4.0, 5.0$; kurtosis=4.34,5.20,6.13,8.01). **f-j** for λ_{Var}; **k-o** for $\widehat{\sigma_\varepsilon}^2$; **p-t** for $\mathrm{Var}[X]$. Red crosses indicate the mean position of the scatter plot in each panel. The concentration of points above the diagonal identity line for λ_{AC1} indicates that its values decline substantially as kurtosis increases, confirming that λ_{AC1} is more sensitive to outlier magnitude than λ_{Var}. By contrast, λ_{Var} remains relatively stable with increasing outlier severity. This robustness arises from its formulation in Eq. S2: although both $\widehat{\sigma}_\varepsilon^2$ and $\mathrm{Var}[X]$ increase under stronger outliers, their ratio remains comparatively unchanged.

Time series from evergreen broadleaf forests exhibit an upper-concentrated pattern, whereas those from closed shrublands display a symmetric pattern in MODIS NDVI datasets. This phenomenon was previously reported by Ref. (51). Figure S14 presents a similar analysis to Figure 2A and B in Ref. (51), yielding comparable results, aside from minor numerical differences due to the use of a stratified random sample.

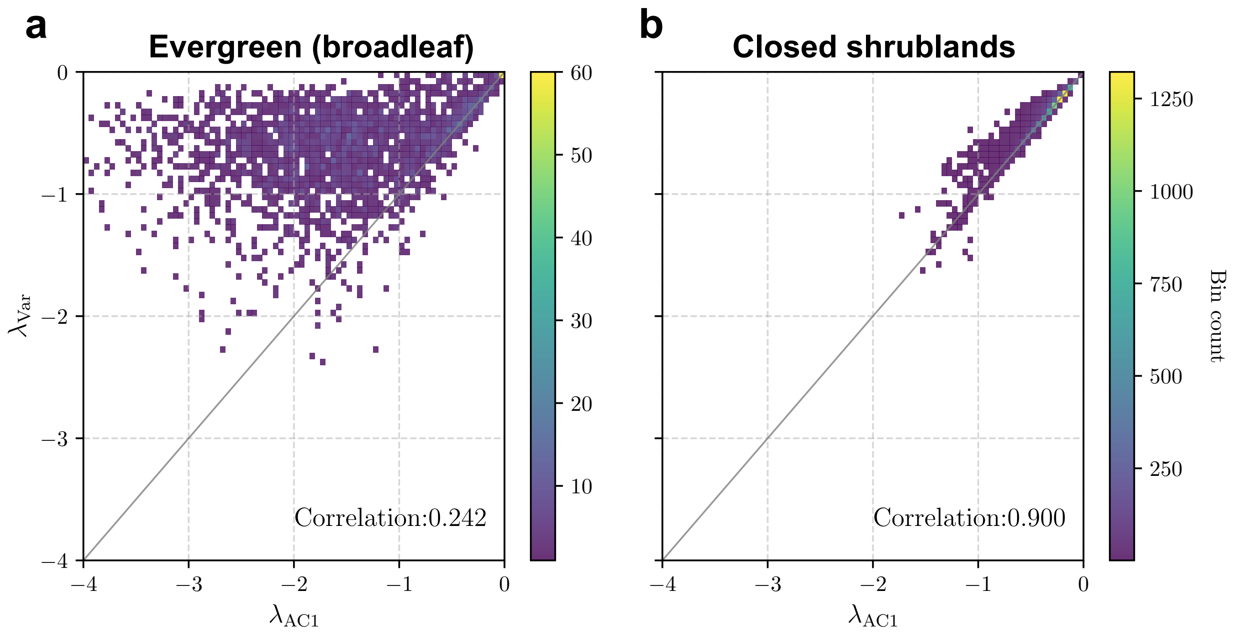

Figure S14: **Relationship between λ_{Var} and λ_{AC1} using NDVI data at MODIS-native resolution (250 m). a**, Evergreen broadleaf (n = 10,000). **b**, Closed shrublands (n = 10,000).

Beyond the number of missing values, their distribution also influences resilience indicators. To demonstrate this effect, we consider two extreme cases: (1) Non-consecutive Gaps (Scattered Missing Intervals): Missing values occur as isolated intervals, ensuring that no two consecutive time points are missing. (2) Consecutive Gaps (Block Missing Interval): Missing values form a single continuous segment, where each missing data point is directly followed by another.

We compared the effects of these two distribution patterns on resilience indicators. As shown in Figure S15, non-consecutive gaps reduce the agreement between λ_{Var} and λ_{AC1} more significantly than consecutive gaps. This can be understood by considering how missing values segment the time series into multiple smaller, complete subseries. For each subseries, the agreement between λ_{Var} and λ_{AC1} is determined by its initial data point. When the time series is divided into numerous subseries, the variation among initial data points increases, leading to greater deviation in overall $\lambda_{\text{Var}} - \lambda_{\text{AC1}}$ agreement. Consequently, the number of subseries directly influences the agreement of λ_{Var} and λ_{AC1} derived from the full time series. In the case of non-consecutive gaps, scattered missing values fragment the time series into numerous short subseries, leading to a substantial reduction in indicator agreement. By contrast, consecutive gaps divide the time series into only two segments, resulting in a smaller impact on the agreement between λ_{Var} and λ_{AC1}.

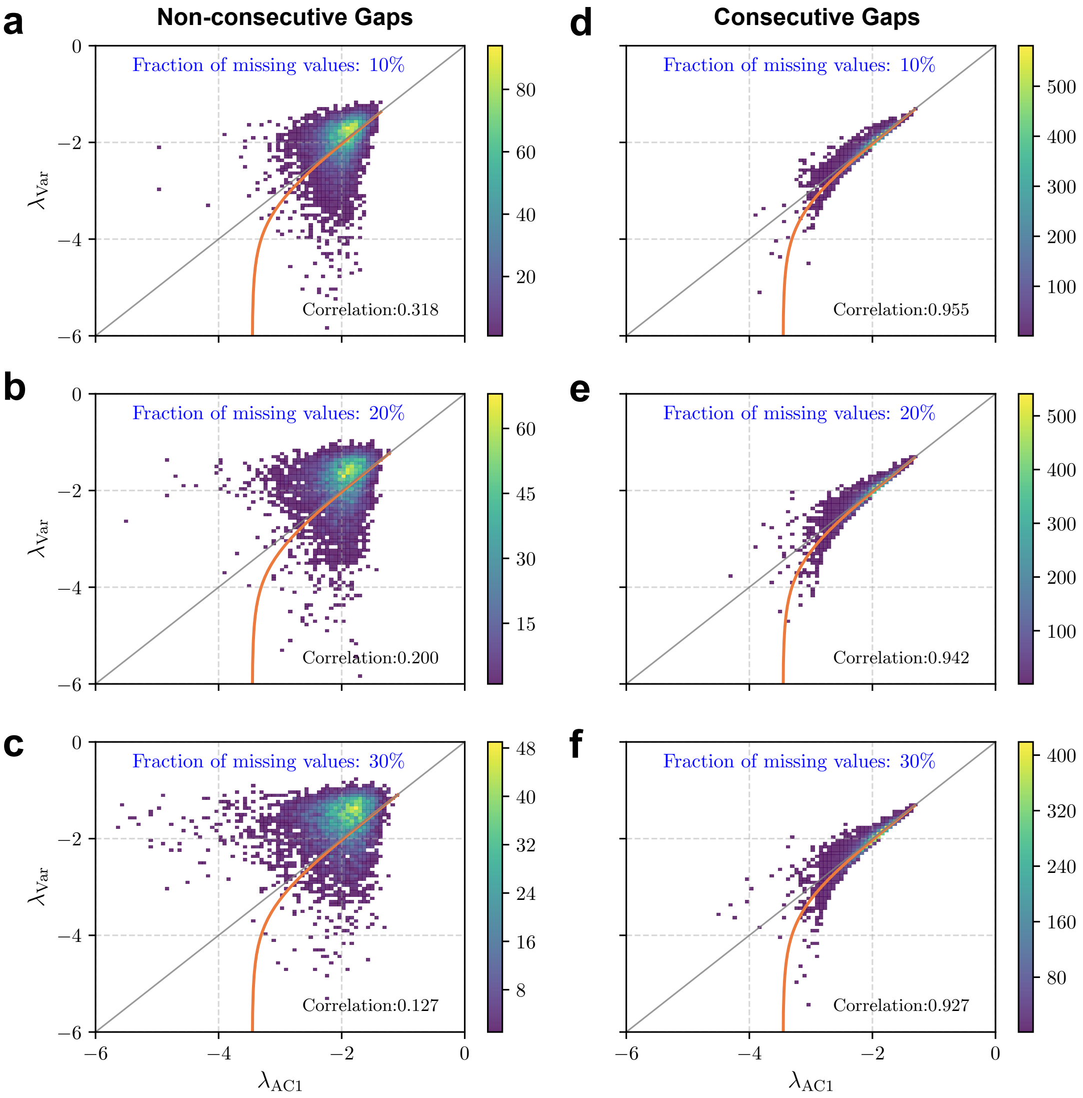

Figure S15: **Relationship between λ_{Var} and λ_{AC1} using simulated time series with different distributions of missing values.** Each condition consists of n = 10,000 time series generated from an AR(1) process, each with a length of 1,000. Non-consecutive gaps were introduced with missing value fraction of 10%, 20%, and 30% (**a-c**), while consecutive gaps were introduced with missing value fraction of 10%, 20%, and 30% (**d-f**).

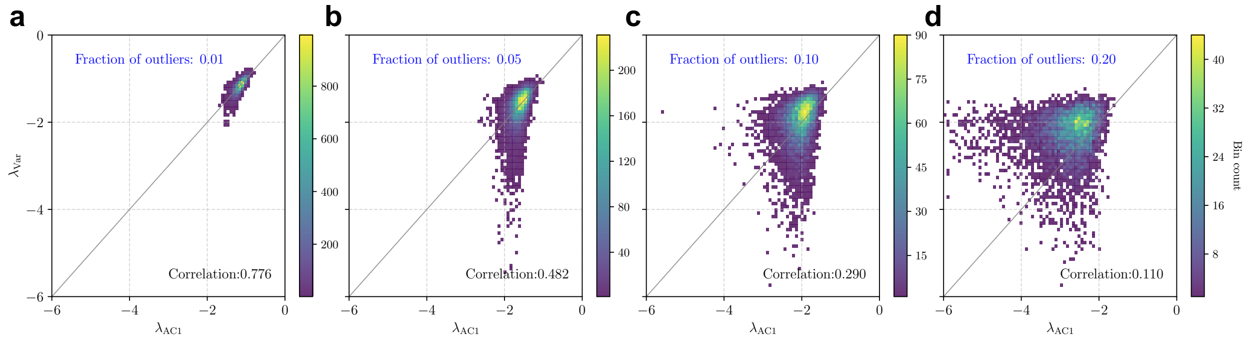

Figure S16: **Relationship between λ_{Var} and λ_{AC1} under varying numbers of outliers.** Each panel presents results from n = 10,000 simulated time series (length = 1,000), generated from an AR(1) process with parameters $\alpha = e^{-1}$ and $\sigma_{\varepsilon} = 1$. Ten percent of values are randomly set as missing. Outlier magnitude is fixed at $k = 3$. The fraction of outliers increases progressively from **a** to **d**, ranging from 1% to 20%. As the outlier ratio increases, the agreement between λ_{AC1} and λ_{Var} systematically declines.
